# Mechanisms of Silver Nanoparticle Uptake by Embryonic Zebrafish Cells

**DOI:** 10.3390/nano11102699

**Published:** 2021-10-13

**Authors:** Ana C. Quevedo, Laura-Jayne A. Ellis, Iseult Lynch, Eugenia Valsami-Jones

**Affiliations:** School of Geography, Earth and Environmental Sciences, University of Birmingham, Edgbaston, Birmingham B15 2TT, UK; L.A.Ellis@bham.ac.uk (L.-J.A.E.); E.ValsamiJones@bham.ac.uk (E.V.-J.)

**Keywords:** nanoparticle uptake, in vitro, silver nanoparticles, toxic effects, nanosafety

## Abstract

Evaluation of the uptake pathways in cells during exposure to nanoparticles (NPs) is key for risk assessment and the development of safer nanomaterials, as the internalisation and fate of NPs is linked to their toxicity and mode of action. Here, we determined the uptake mechanisms activated during the internalisation of 10, 30, and 100 nm AgNPs by embryonic zebrafish cells (ZF4). The uptake results demonstrated an NP size- and time-dependent uptake, showing the highest total silver uptake for the smallest AgNP (10 nm) at the lowest exposure concentration (2.5 μg/mL) after 2 h, while after 24 h, the highest exposure concentration (10 μg/mL) of the 10 nm AgNPs revealed the highest cellular load at 8 pg/cell. Inhibition of the caveolae, clathrin, and macropinocytosis endocytic pathways by pharmaceutical inhibitors (genistein, chlorpromazine, and wortmannin respectively) revealed that uptake was mainly via macropinocytosis for the 10 nm AgNPs and via the caveolae-mediated pathway for the 30 and 100 nm AgNPs. The induction of autophagy was also strongly related to the NP size, showing the highest percentage of induction for the 10 nm (around 3%) compared to naive cells, suggesting that autophagy can be activated along with endocytosis to deal with exposure to NPs. TEM imaging revealed the distribution of NPs across the cytoplasm inside intracellular vesicles. An increase in Early Endosome formation (EE) was observed for the 30 and 100 nm sizes, whereas the 10 nm AgNPs disrupted the activity of EE. The data supports the establishment of adverse outcome pathways by increasing knowledge on the link between a molecular initiating event such as receptor-mediated endocytosis and an adverse outcome, as well as supporting the reduction of animal testing by using alternative testing models, such as fish cell lines.

## 1. Introduction

Silver nanoparticles (AgNPs) are broadly used in the biomedical and industrial fields and are being incorporated into many daily life products, such as paints and textiles, due to their highly effective antimicrobial properties [1,2]. NPs’ physicochemical characteristics, such as small size and large surface area, provide numerous advantages in hundreds of nano-based products; however, negative effects also arise from these unique characteristics, demanding proper toxicological assessment of the possible negative outcomes linked to their release into the environment, in conjunction with an in-depth understanding of their biological behaviour and effects following cellular exposure and internalisation [3,4].

Cellular uptake is one of the most important processes regulating the biological activity of cells, involving the use a range of transport mechanisms to move macromolecules and particles from the surrounding medium across the cell membrane by a process called receptor-mediated endocytosis [5,6]. Endocytosis can be broadly divided into phagocytosis and pinocytosis. During phagocytosis, cells (mainly macrophages or dendritic cells) engulf particulate matter to digest it, using receptors on the cell surface for recognition and activation [5,7]. On the other hand, pinocytosis uses vesicles to internalise fluids and other molecules, involving different processes, depending on the molecular mechanism activated. Clathrin or caveolin-dependent endocytosis trigger the production of endocytic vesicles called early endosomes (EE), which accept the incoming cargo internalised from the cell membrane [5]. Caveolae-dependent endocytosis internalises external matter in flask-shaped vesicles, through the binding between receptors on the cell membrane and the external matter [6,8]. Finally, macropinocytosis involves the formation of large vesicles, internalising nonspecific cargo and large amounts of fluid [6,9]. Autophagy, while not fully recognised as an endocytic pathway, plays an important role in cellular internalisation due to its physiological role in engulfing, transporting, and degrading internalised cargo [10].

The cellular uptake of NPs involves highly regulated mechanisms that are interrelated with other complex biomolecular interactions [11]. The use of pharmaceutical inhibitors to assess the endocytic pathways involved in the uptake of NPs has been widely accepted in the scientific community and applied in a variety of cellular models [8,12,13,14]. The effectiveness of pharmacological inhibitors is determined through assessment of the reduction of uptake of a chemical known to be internalised through that specific endocytic pathway, demonstrating the effectiveness of the inhibitor to block the desired pathway and the assay performance [8]. The interactions between NPs and the environment imposes challenges for their characterisation as a result of physical and chemical transformations and the required determination of outcomes at the cellular and subcellular levels in relevant aquatic models and under relevant exposure conditions [11,15,16].

Zebrafish have been used for many years as a regulatory test species and has been validated by the Organisation for Economic Cooperation and Development (OECD); however, their use requires an animal handling license and ethical approval. Due to these difficulties, the use of zebrafish embryos is considered a more viable option for assessing acute toxicity without requiring ethical approval (up to 5 days post fertilisation) [17]. Despite the numerous benefits embryos present in the nanotoxicological field, some of their characteristics, such as the protective chorion, may compromise the assessment of NP-induced biological responses, as the chorion may represent a barrier between NPs and their internalisation. On the other hand, chorion removal involves complex processes with non-environmental relevance, resulting in inhibition of the hatching process, low survival rate, and sensitivity [18,19,20].

The use of fish cells represents a feasible option for fast toxicological screening, including assessment of the mechanisms implicated in the internalisation, distribution, and (adverse) effects of NPs. As an aquatic model, it also has advantages in terms of avoiding the ethical challenges associated with sentient organisms, supporting the transition to in vitro testing. In fact, zebrafish cells, such as embryonic zebrafish cells (ZF4) and zebrafish liver (ZFL) cells, have gained popularity in past years as a potential early-stage model, mainly for the assessment of toxicological and biological responses, including translational gene expression [21], bacterial invasion [22], assessment of pharmacological chemicals [23], and impacts from polystyrene [24,25] and metallic NPs [26,27,28]. To date, only a few studies have explored the trafficking and uptake mechanisms of nanomaterials in zebrafish cells, highlighting a key knowledge gap that needs to be urgently explored [24,25,26,28].

In the present study, we investigated endocytic and non-endocytic (autophagy) mechanisms involved in the internalisation of cargo by ZF4 cell aiming to determine which pathways were utilised for the uptake of different sizes (10, 30, and 100 nm) and concentrations of AgNPs, through the use of pharmacological inhibitors. Our ultimate aim was to demonstrate the utility of the ZF4 adherent continuous cell line for assessment of nanotoxicity, in support of the replacement, reduction, and refinement of animals in experimentation through the 3Rs framework [29,30]. Furthermore, ZF4 cells can bridge the gap between chemical analysis and biological responses by assisting in the development of adverse outcome pathways (AOP), which focus on gaining information about the initial interaction of xenobiotics within a biological systems (the molecular initiating event), assessing the series of intermediate events that will eventually lead to an adverse outcome [30].

## 2. Materials and Methods

### 2.1. Characterization of the AgNPs

Polyvinylpyrrolidone (PVP) coated silver nanoparticles (AgNPs) with sizes of 10, 30, and 100 nm were purchased from Nanocomposix, USA. The characterisation of AgNPs was performed in different testing media, such as the simple water matrix Ultrapure Water (UPW) and Complete Culture Media (CCM), prepared with the cell medium (DMEM/F12,11330, Gibco, Amarillo, TX, USA) supplemented with 10% Foetal Bovine Serum (FBS; 10270, Gibco, Amarillo, TX, USA) and 1% penicillin and streptomycin (15070, Gibco, Amarillo, TX, USA). Dynamic light scattering (DLS) (Zetasizer Nano series, Malvern Panalytical, Great Malvern, UK) was used to assess the hydrodynamic size, polydispersity index (PDI), and zeta potential. Ultraviolet visible light (UV–Vis) spectrophotometry was used to determine the optical spectra of the AgNPs (Abs_max_) and to provide a complementary measure of particle size.

Both techniques were applied in UPW and CCM, with AgNP dispersions prepared at a final concentration of 10 μg/mL and incubated at 28 °C for 0 and 24 h. For size and PDI measurements, the suspensions were placed in disposable polystyrene cuvettes (Sarstedt, Newton, NC, USA, 67.742) at a total volume of 1 mL; for zeta potential measurements, the sample was prepared in a total volume of 700 μL and placed in a folded capillary cell (Malvern, DTS1070); and finally, for UV–Vis measurements, 1 mL of the sample was placed in 3 mL disposable cuvettes (Sarstedt, Newton, NC, USA, 67742). All the samples were prepared fresh and immediately evaluated. Transmission electron microscopy (TEM) samples were prepared in UPW to assess the core size of the NPs. The TEM sample preparation is described in a previous publication [27]. Briefly, samples with a concentration of 100 μg/mL were prepared fresh, then 15 μL of the AgNPs suspension was loaded onto a copper grid, and after 2 h the sample was gently washed with UPW and left to dry for 24 h.

### 2.2. Culturing of ZF4 Cells

Embryonic zebrafish (ZF4) cells were cultured as described on the manufacturer’s website (https://www.atcc.org/products/all/CRL-2050.aspx, accessed on 15 October 2021) and in previous publications [26,27]. Briefly, cells were cultured in T75 flasks with a vented cap (Corning, Corning, NY, USA, 430641U) and in CCM. The CCM was prepared with DMEM/F12, supplemented with 10% FBS, 1% penicillin, and streptomycin, and incubated in a humidified atmosphere of 5% CO_2_ at 28 °C. Cells were passaged upon reaching 80% confluence (Passage 28) by discarding the cell medium and gently washing with 5 mL of warm phosphate-buffered saline (PBS) (Thermofisher, Waltham, MA, USA, D5837). Then, cells were detached using 1.5 mL of 0.25% trypsin (Gibco, Amarillo, TX, USA, 15090) for 3 min at 28 °C. To maintain the cell line in T75 flasks, 2 mL of the diluted cell suspension was transferred into a new flask, diluted with 8 mL of CCM, and left to grow for one week with 5% CO_2_ at 28 °C. For large experiments, 3 mL of the detached cells were transferred to T175 vented cap flasks (Corning, Corning, NY, USA, 431080), diluted with 17 mL of CCM, and left to grow for one week before seeding in well plates for experiments. To maintain the cell line in T175 flasks, cells were washed with 10 mL of PBS and detached with 3 mL of trypsin for 3 min, then 2 mL of the cells were re-seeded in T175 flasks, diluted with 18 mL of CCM, and left to grow for one week.

### 2.3. Imaging of Intracellular AgNPs by TEM

To confirm the presence of the particulate form (AgNPs) and to provide evidence of the endocytosis pathways activated in ZF4 cells exposed to AgNPs, TEM analysis of the highest AgNPs concentration (10 μg/mL) was performed for each AgNP size. The protocol described by Ellis et al. (2020) was modified and adapted to ZF4 cells [31]. Briefly, ZF4 cells were seeded in six-well flat bottom plates (Corning, Corning, NY, USA, CLS3736) at a density of 5 × 10^5^ cells in a total volume of 2 mL per well, 24 h prior to the study. After 24 h, cells were treated with 10 μg/mL of the 10, 30, and 100 nm AgNPs for 24 h at 28 °C. After the incubation period, the cell medium was removed, and cells were washed with 1 mL of warm PBS and then detached using 0.25% trypsin for 3 min at 28 °C in a total volume of 2 mL. The detached cells were centrifuged for 10 min at 270× *g* at 20 °C (Eppendorf, Hamburg, Germany, 5430R). Then, the supernatant was carefully removed, and the cell pellet was diluted with 2.5% glutaraldehyde in 0.1 M PBS provided by the Centre for Electron Microscopy at the University of Birmingham (UK), followed by 10 min of centrifugation at 270× *g* at 20 °C. Finally, cells were dehydrated in ethanol and embedded in epoxy resin before sectioning using an ultramicrotome to cut 0.1 μm sections with a diamond knife. Sections were collected onto 300 mesh copper (Cu) grids on carbon film (Agar Scientific, Stansted, UK, AGS160-3) and images were visualised using JEOL 1200EX 80 kV and JEOL 1400EX 80 kV microscopes (JEOL, Tokyo, Japan).

### 2.4. Visualisation of the Inhibition of Cellular Uptake Pathways by Confocal Microscopy

When using inhibitors for endocytosis pathways, it is essential to confirm that the desired uptake pathway was effectively inhibited by the respective chemical inhibitor in order to obtain reliable and representative results. To test the inhibition of the desired cellular uptake pathways, ZF4 cells were seeded in 24 well MatTek 13 mm glass-bottom dishes (MatTek, Ashland, MA, USA, P24G013F) at a density of 100,000 cells/mL in a total volume of 1 mL per well, with DMEM/F12 supplemented with 10% FBS, and 1% of penicillin and streptomycin at 28 °C and 5% CO_2_. Twenty-four hours post-seeding, cells were pre-incubated with different concentrations of chemical inhibitors determined based on literature review [6,8,14], selecting the following as the final concentrations which successfully inhibited the desired uptake pathway: 100 μg/mL of genistein (G6649, Sigma, St. Louis, MO, USA) for 20 min at 28 °C to inhibit the caveolae pathway, 10 μg/mL of chlorpromazine (C8138, Sigma, St. Louis, MO, USA) for 30 min at 28 °C to inhibit the clathrin- mediated pathway, and 10 μg/mL of wortmannin (W1628, Sigma, St. Louis, MO, USA) for 10 min at room temperature for the macropinocytosis pathway.

All the inhibitors were prepared at the desired concentration in CCM in a total volume of 500 μL per well. After the incubation period with the chemical inhibitors, the cell medium was removed and the cells were carefully washed with 500 μL of warm PBS, then the respective markers (known to selectively enter cells via a specific pathway) were added for 2 h at 28 °C (in a total volume of 500 μL) as positive controls to confirm that the inhibition was successful. The final concentrations for the transport molecules (controls) were as follows: 1 μg/mL of cholera toxin b (C1655, Sigma, St. Louis, MO, USA) for genistein treatments, 500 μg/mL of Transferrin Conjugate Alexa FluorTM 488 (T13342, ThermoFisher, Waltham, MA, USA) for chlorpromazine treatments, and 500 μg/mL of dextran-Rhodamine B (D1824, ThermoFisher, Waltham, MA, USA) for wortmannin treatments. After the incubation time, the cell medium was removed, and cells were washed twice with warm PBS. Then, cells were fixed for 20 min with 500 μL of 4% Paraformaldehyde (16% Thermofisher, Waltham, MA, USA, 43368) diluted in PBS and then examined by confocal microscopy using a NIKON A1R 808 series microscope (Nikon, Tokyo, Japan). Images were recorded with a 60× objective lens; the red filter was used for dextran-rhodamine B, which has an Excitation/Emission of 647⁄668 nm; the green filter was used for cholera toxin b and transferrin conjugate Alexa Fluor both with an Ext/Em of 495/519 nm. Images were processed using the FIJI open-source image processing tool (V.2.00-RC69, National Institutes of Health, Bethesda, Maryland, MD, USA).

### 2.5. Quantification of Ag^+^ Uptake by ZF4 Cells by ICP–MS

To assess the uptake of AgNPs in ZF4 cells by Inductively Coupled Plasma Mass Spectrometry (ICP–MS), different methodologies were tested to standardise the protocol to ensure the maximum detection of ionic silver [32,33,34]. The final protocol was established as follows. First, ZF4 cells were seeded in six-well flat bottom plates (Corning, Corning, NY, USA, CLS3736) at a density of 5 × 10^5^ cells per well in a total volume of 2 mL, using DMEM/F12 supplemented with 10% FBS and 1% of penicillin and streptomycin at 28 °C and 5% CO_2_. At 24 h post-seeding, ZF4 cells were treated to a low, medium, and high concentration (2.5, 5, and 10 μg/mL as determined in our previous studies using the lactate dehydrogenase (LDH) cytotoxicity assay [23]) of the three representative AgNPs sizes (10, 30, and 100 nm); then, cells were incubated for 24 h at 5% CO_2_ and 28 °C.

After the 24 h incubation period, the cell medium was removed, and 3 mL of aqua regia was added. The aqua regia was prepared with 37% hydrochloric acid (H/1200/PB17, Fisher Scientific, Hampton, NH, USA) and 70% nitric acid (A509-P500, TraceMetal^TM^, Fisher Scientific, Hampton, NH, USA) in a ratio of 3:1. Cells attached to the six well plates were carefully washed with 500 μL of warm PBS twice to ensure the removal of non-intracellular AgNPs. Cells were detached using 0.25% trypsin for 3 min at 28 °C. Then, 3 mL of aqua regia (prepared as previously described) were added, then cells were resuspended and transferred to a clean glass vial. The glass vials were properly closed and placed in an oven overnight at 70 °C. The next day, the acid digested samples were diluted with ultra-pure water to reach a 2% HNO_3_ concentration. Then, the diluted samples were filtered with 22 μm syringe filters (E4780-1226, StarLab, Brussels, Belgium) to ensure the full extraction of only ionic silver. Finally, the filtered sample was analysed by ICP–MS (NexION 300×, Perkin Elmer, Waltham, MA, USA). A calibration curve of silver plasma emission standard (Cat. No. 456892C) prepared in 2% HNO_3_ was used to setup the ICP–MS. Three individual replicates were included for each AgNP size and concentration.

### 2.6. Quantification of Ag^+^ by ICP–MS after Inhibition of the Cellular Uptake Pathways

Having confirmed the relevant concentrations of the pharmacological inhibitors by confocal microscopy (see above), ZF4 cells were seeded in six-well flat bottom plates (Corning, Corning, NY, USA, CLS3736) at a density of 5 × 10^5^ cells per well in volume of 2 mL, using DMEM/F12 supplemented with 10% FBS and 1% of penicillin and streptomycin at 28 °C and 5% CO_2_. After 24 hrs, cells were incubated with 100 μg/mL of genistein for 20 min at 28 °C, 20 μg/mL of chlorpromazine for 30 min at 28 °C, and 10 μg/mL of wortmannin for 10 min at room temperature. Inhibitors were prepared in CCM for a total volume of 2 mL per well. After incubation with the chemical inhibitors, the cell medium was discarded, and cells were treated with 2.5, 5 and 10 μg/mL of AgNPs for 10, 30, and 100 nm sizes for 2 h at 28 °C in a final volume of 2 mL per well. Finally, the cell culture medium was removed; then, cells attached to the six well plates were washed, acid digested, diluted, and analysed by ICP–MS, as described in Section 2.5. Three individual replicates were analysed for each NP size, concentration, and chemical inhibitor used.

### 2.7. Detection of Early Endosome (EE) Formation by Fluorescence Measurements

To further understand the endocytosis process, the induction of EE was evaluated by labelling early endosomes with red fluorescent protein (RFP). Cells were transduced with cellLightTM Early Endosomes-RFP BacMam 2.0 (Cat. No. C10587; Thermofisher, Waltham, MA, USA)) in accordance with the manufacturer’s instructions. Briefly, ZF4 cells were seeded in 96-well flat bottom plates (Corning, Corning, NY, USA, Cat. No. 3917) at 8000 cells per well and in a final volume of 200 μL. Cells were left to attach overnight at 28 °C with 5% CO_2_. The next day, AgNP treatments of 2.5, 5, and 10 μg/mL for different AgNP sizes (10, 30, and 100) were mixed with 2 μL of BacMam 2.0 reagent (Thermofisher, Waltham, MA, USA) in a final volume of 200 μL per well. Then, the cell medium was gently aspirated, and cells were treated with the AgNPs mixed with the cell light dye and incubated for 24 h at 28 °C with 5% CO_2_. After the incubation time, the intensity of the red fluorescent protein of three individual replicates was measured by fluorescent microplate reader (Spark by Tecan, Männedorf, Switzerland) using an excitation and emission of 555/584 nm, respectively. Fluorescence intensity results were normalised to percentage (%) against untreated cells (naive), indicated as *N*%.

$$N\%=\frac{Intensity\;\left(each\;value\right)}{mean\;naive\;}*100$$

### 2.8. Autophagy Response

To evaluate the autophagy response involved in the uptake of AgNPs by ZF4, a modified protocol for autophagy evaluation by flow cytometry was adapted for confocal microscopy [27]. Briefly, ZF4 cells were seeded in uncoated 24 well MatTek 13 mm glass- bottom dishes (MatTek, Ashland, MA, USA, Cat. No. P24G013F) at a density of 100,000 cells per well in a final volume of 1 mL with CCM, and incubated overnight at 28 °C and 5% CO_2_. 24h post-seeding, cells were treated with 2.5, 5, and 10 μg/mL of 10, 30, and 100 nm AgNPs for 24 h. After the incubation period, the cell medium was removed, and 500 μL of CCM mixed with nucleus and lysosome staining was added to the cells and incubated at 28 °C for 30 min. The organelle labelling solution was prepared in warm CCM containing 1 μL/mL of Hoechst 33342 (Thermofisher, Waltham, MA, USA, Cat. No. 62249) to stain the nuclei and 1 μL/mL of LysoTracker^TM^ Deep Red (Thermofisher, Waltham, MA, USA, Cat. No. L12492). After the incubation period, the medium containing the first dyes was removed, and cells were washed twice with warm PBS. Then, cells were stained using a Cell Meter^TM^ Autophagy Assay Kit (Cat. No. 23002; Sunnyvale, CA, USA). First, a stock solution was prepared with 20 μL of the Autophagy Green^TM^ diluted in 10 mL of Stain Buffer. Then, 500 μL of the staining solution was added to cells (per well) and incubated at room temperature for 30 min in the dark. Then, the autophagy staining medium was removed, and cells were washed with warm PBS once. Immediately a fixation was performed with 500 μL of 4% Paraformaldehyde (16% Thermofisher, Waltham, MA, USA, Cat. No. 43368) diluted in PBS. Finally, cells were examined by confocal microscopy using a NIKON A1R 808 series microscope (Nikon, Tokyo, Japan). Images were recorded with a 60× objective lens for all channels. For nucleus identification, the blue laser was used, with an excitation and emission of 350⁄461 nm; the red filter was used for the lysosomes (647⁄668 nm); and for autophagy response, the green filter (495/519 nm) was used. The intensity of three individual cells per replicate (n = 3) were analysed by the FIJI open-source image processing tool (V.2.00-RC69, National Institutes of Health, Bethesda, Maryland, MD, USA) as described in Quevedo et al., 2021.

### 2.9. Protein Corona Isolation and Analysis of Proteins by Polyacrylamide Gel Electrophoresis (PAGE)

To further understand the implications of the NP–protein interactions, the protein coronas acquired by the incubation of the AgNPs in CCM alone and CCM conditioned with cells attached to the flask were isolated and analysed by mass spectrometry, following the protocol described by Monopoli et al., (2013) [35]. The full methodology for the incubation and extraction approaches, and the PAGE analysis, are described in Section S7 in the Supplementary Materials. Briefly, after exposure to the NPs, the cell medium containing the coronas was removed, then a series of centrifugation and washing steps were performed to remove unbound proteins by centrifugation in PBS at 20,073× *g* (Eppendorf, Hamburg, Germany, 5430R) for 20 min. Then, the sample pellet was re-suspended and incubated at 95 °C for 5 min and centrifugated again at 20,073× *g* for 30 min. The pellet was diluted with sodium dodecyl sulphate (SDS) and stored at −20 °C for further analysis. The isolated proteins were run on a 12.5% polyacrylamide gel electrophoresis (PAGE) and stained with 25% Coomassie blue. Bands confirming the presence of proteins were sent to the Advanced Mass Spectrometry Facility in the School of Biosciences at the University of Birmingham for analysis. The proteins secreted by the ZF4 cells during the exposure to AgNPs were also evaluated by quantification of the total protein concentrations in the media using a BCA protein assay kit (ThermoFisher, Waltham, MA, USA, 23225).

### 2.10. Statistical Analysis

The results were normalised against the control for each experiment and then plotted and statistically analysed using GraphPad V8.1 software (GraphPad Software, San Diego, CA, USA) by one or two-way ANOVA, followed by a Bonferroni post-hoc multiple comparison for all the AgNPs treatments against the untreated control (naïve), unless otherwise stated in the figure legend.

## 3. Results

### 3.1. Characterisation of the AgNPs

**Figure 1:**
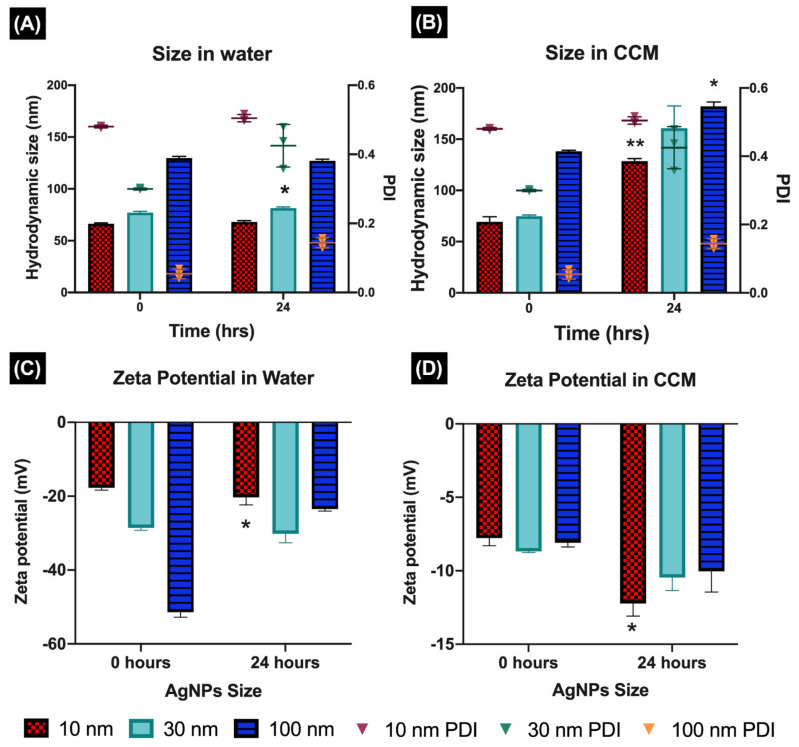
Characterisation of AgNPs. The characterisation of the AgNPs was performed following 0 and 24 h of incubation in water and CCM by DLS. (**A**,**C**) show the hydrodynamic size and zeta potential in water. (**B**,**D**) show the hydrodynamic size and zeta potential in CCM. The A and B left axes show the PDI recorded for each AgNPs sample. Data with asterisks * indicate statistical differences (* *p* < 0.05 and ** *p* < 0.01) between timepoints (0 and 24 h) for the selected NP size.

The characterisation of the AgNPs in a simple testing media such as UPW and in more complex and relevant environments (CCM) are key to understanding NP impacts on biological systems. The core size was evaluated by TEM in UPW; the results demonstrated that the core was very close that stated by the manufacturer, with recorded sizes of 13 ± 2.4 for the 10 nm, 34 ± 2.8 for the 30 nm, and 101.6 ± 9.2 for the 100 nm (see the publication Quevedo et al., 2021 and Supplementary Materials Figure S1 in the Supplementary Information (SI) for representative TEM images). The hydrodynamic size in UPW determined by DLS revealed that all three AgNPs sizes remained stable between 0 and 24 h, showing a small increase of 2–3% between time points. The polydispersity index (PDI) presented no changes for 10 and 30 nm at the different time points, whereas the 100 nm displayed a small decrease in PDI after 24 h (from 0.06 to 0.03). The zeta potential in water displayed similar values between time points for the 10 and 30 nm, with less than 2% variation. On the other hand, the 100 nm AgNPs displayed a noticeable decrease in zeta potential after 24 h, with a final value of −23.46 ± 0.56 mV, despite the particle stabilisation being steric in nature as result of the PVP coatings (Figure 1C).

The AgNPs hydrodynamic size after 24 h in CCM was higher than in water, as expected, due to the presence of proteins and other biomolecules, which influence the dynamic behaviour of AgNPs in complex environments. The 10 and 30 nm sizes increased approximately 50% after 24 h (Figure 1B), compared to their initial size. In contrast, the 100 nm showed the lowest increase in hydrodynamic size, reaching 182.06 ± 4.25 nm after 24 h. The values for the zeta potential in CCM showed noticeable changes compared to the values in water (became less negative) due to the presence of FBS in the cell culture medium, which produced a shielding effect, charge neutralization, and bridging interactions with the serum proteins. For example, the zeta potential values in CCM ranged between −7 and −12 mV at 0 and 24 h, compared to values in water, which ranged from −17 and −57 mV at both time points (Figure 1D).

The absorption spectra (Abs_max_) assessed by ultraviolet visible light (UV–VIS) spectrophotometry also displayed differences between tested media. Samples in UPW displayed higher absorption efficiency, as the absorption dominates UV–VIS spectra, displaying only one peak for each measurement, whereas the samples in CCM showed two peaks and scattered photon flux due to the presence of proteins and other biomolecules in the cell medium (Supplementary Materials Figure S1 in the SI). The 10 and 30 nm NPs showed major changes in the Abs_max_ at both timepoints, whereas the 100 nm showed only minor differences in absorbed light between measurements, with around <1%. The samples prepared in CCM revealed different trends for each AgNP size due the protein background. The 10 nm presented almost 50% decrease from the initial recorded size after 24 h. The 30 nm size showed almost equal values for both time points (around 1.0 Abs_max_), indicating very limited evolution of the sample beyond the initial interaction with the FBS. The absorbance of the 100 nm AgNPs displayed an increase compared to the initial timepoint, reaching 1.195 ± 0.00 Abs_max_ after 24 h, reflecting light from internal interfaces and creating a scattering component. A summary of all the results, including UV–Vis images, can be found in Supplementary Materials Table S1 and Figure S2 in the Supplementary Materials.

### 3.2. Intracellular Localisation of AgNPs

**Figure 2:**
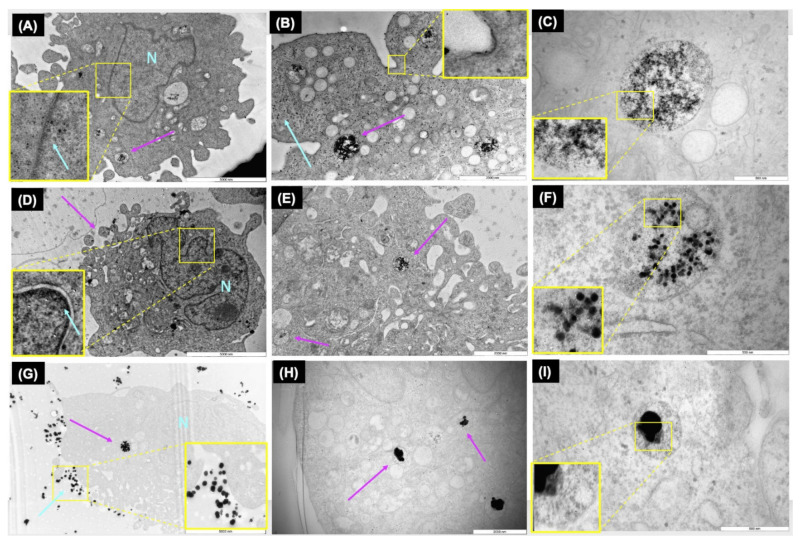
TEM images of intracellular AgNPs. The images show the intracellular fate of 10 µg/mL of 10, 30, and 100 nm AgNPs. (**A**–**C**): uptake of 10 nm AgNPs. The yellow square in B shows an enlargement of the cell’s uptake machinery; (**D**–**F**): uptake of 30 nm AgNPs; and (**G**–**I**): uptake of 100 nm AgNPs. The scale bars correspond to 5000, 2000, and 500 nm from left to right. Purple arrows indicate the formation of vesicles in the cells, blue arrows indicate NPs in the zoomed figures (yellow squares), and N means nucleus.

Transmission Electron Microscopy (TEM) is a valuable tool for the morphological characterisation of biological and nonbiological materials at high resolution. Hence, to confirm the internalisation of AgNPs (nanoparticulate form) in ZF4, as well as to explore possible changes NPs may induce following contact with ZF4 cells, the highest AgNP concentration (10 μg/mL) was analysed by TEM. Images confirmed the uptake and localisation of AgNPs, demonstrating the internalisation of the AgNPs in the intracellular vesicles that engulfed them. Furthermore, the nucleus can be identified, and the images indicate that small and medium NPs were located in close proximity to the nucleus (see magnifications in Figure 2A,D). The 10 nm AgNPs inside the intracellular vesicles showed signs of density loss, potentially from dissolution, which made assessment of their diameter challenging (Figure 2C). Despite this, the diameter of the intracellular NPs was recorded as 5.50 ± 3.56 nm, compared to the core size of the NPs determined by TEM (13 ± 2.4 nm) (see Supplementary Materials Figure S1 in the SI for TEM images), resulting in a size reduction of 57.62% (Figure 2A–C). The diameter of the vesicles with encapsulated NPs displayed a size of 717.46 ± 78.57 nm (mean ± SD of three different vesicles). This supports a possible macropinocytosis uptake pathway, mainly through the formation of large vesicles in the cell membrane (0.15 to 5.0 μm in diameter), which can mediate the uptake of molecules (for details about the analysis of the vesicles, see Section 3 and Supplementary Materials Figure S3 in the SI) [7]. Furthermore, the TEM images revealed a possible caveolae pathway as shown in the yellow box in Figure 2B. The internalisation of the 30 nm AgNPs is presented in Figure 2D–F. Figure 2E shows the uptake of AgNP by endosomes as well as part of their subcellular trafficking in the cell (purple arrows); the localisation of the vesicles within the cellular matrix is likely related to late endolysosomes. The larger magnification in Figure 2D reveals that 30 nm AgNPs are located inside a vesicle and very close to the nucleus (N). The diameter of the intracellular vesicles for the 30 nm size was measured as 697.86 ± 53.76 nm. Analysis of the size of the AgNPs inside the vesicles revealed a size of 26.37 ± 4.55 nm and a percentage size reduction of 19.04% compared to the original TEM size of 34 ± 2.8 nm. The reduction in size can also be related to the fragmentation of the NPs by the digestive complexes inside the endosomes, as well as partial dissolution of the NPs, as seen in Figure 2F.

TEM images for the 100 nm AgNPs showed a similar pattern, with a clear internalisation of the NPs inside vesicles as shown in Figure 2G–I. Figure 2G,H showed a large number of NPs outside the cellular membrane (blue arrow). Inside the cellular matrix, encapsulated in endosomes, a large cluster of AgNPs is visible, with signs of fragmentation or dissolution (Figure 2I). The size of the intracellular NPs in the vesicles was calculated as 96.78 ± 8.36, with the lowest percentage of reduction (4.12%) of the three sizes, compared to the initial size of 101 ± 9.2 nm. Overall, the TEM images illustrate that ZF4 cells activate endocytosis mechanisms in response to the AgNPs, which will be further confirmed in inhibition results. The images revealed that the cells initiate uptake pathways by forming cell membrane invaginations (vesicles) that engulf the cargo (NPs), separating from the cell membrane to transport and deliver the cargo into the lysosomes for their degradation. The results for the calculated intracellular NP sizes can be found in Supplementary Materials Table S2 in the Supplementary Materials.

### 3.3. Cellular Uptake of Ag by ICP–MS

The total content of Ag^+^ in ZF4 cells following exposure to the AgNPs was evaluated by ICP–MS. The mass results (μg/mL) obtained by ICP–MS analysis were re-calculated as Ag^+^/cell based on the cell viability (no. cells) recorded by a previous viability assay (LDH assay) at the same AgNP concentrations (2.5, 5 and 10 μg/mL), and from a total number of 8000 cells seeded per 96 wells. Further details about the LDH methodology refer to Section 2.1 in the SI and our previous publication [26]. Then, for easier interpretation, the Ag^+^/cell results were normalized using an equal cell number of 100,000 cells, and finally transformed to pg/cell. (Recalculation results can be found in Supplementary Materials Table S3 in the SI.)

**Figure 3:**
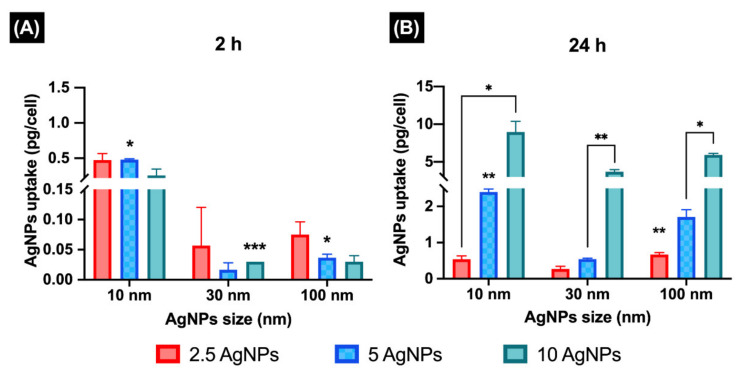
Intracellular uptake of AgNPs. ZF4 cells were treated with 2.5, 5, and 10 µg/mL of three different AgNP sizes (10, 30, and 100 nm) for 2 and 24 h. The total uptake of AgNPs (as Ag^+^) was assessed by ICP–MS. (**A**) Total intracellular Ag^+^ for each concentration and AgNP size after 2 h of exposure. (**B**) Total intracellular Ag^+^ after 24 h. Mass results (µg/mL) obtained by ICP–MS were normalised to total Ag^+^/cell in an equal number of 100,000 cells and then transformed to pg/cell for simpler representation. Three individual replicates were performed for each AgNP concentration, size, and timepoint. Data with asterisks (*) indicate statistical differences (* *p* < 0.05, ** *p* < 0.01, and *** *p* < 0.001) for the NP treatments compared to the untreated cells (naive), whose values (zero) were not included in the graph.

The results for the total Ag^+^ in cells displayed different trends at 2 and 24 h (Figure 3). The normalised results showed that after 2 h, the 10 nm AgNPs presented the highest total Ag^+^ uptake values compared to the other sizes, with similar values (0.47–0.48 pg Ag/cell) at 2.5 and 5 μg/mL, and a statistical difference at the medium concentration (** *p* < 0.01), followed by a decrease at 10 μg/mL (Figure 3A). The 30 nm AgNPs displayed a lower uptake compared to the 10 nm particles, showing a statistically significant difference at the highest concentration (*** *p* <0.001) compared to the untreated control (naive), whereas the low and medium concentrations showed values between 0.05 and 0.01 pg/cell. These results can be related to the fact that cells initiate the uptake processes to deal with low concentrations of NPs, whereas at higher concentrations, the NPs may induce cytotoxic effects, with the cell decreasing uptake processes as it tries to overcome the damage. On the other hand, the 100 nm AgNPs displayed the maximum uptake at the lowest exposure concentration (0.07 ± 0.02 pg/cell), while the 5 and 10 μg/mL displayed identical values, with a statistical difference at 5 µg/mL (* *p* < 0.05) compared to the control (Figure 3A). These results could be linked to the likelihood of large NPs undergoing precipitation and/or aggregation, influencing their uptake.

The uptake after 24 h displayed higher internalised concentrations for all the AgNP sizes, compared to the 2 h exposure. The internalised concentration of the 10 nm AgNPs was 0.54 pg/cell for the lowest concentration (2.5 µg/mL), and with the highest exposure concentration (10 µg/mL), resulting in a statistically significant (* *p* < 0.05) total Ag^+^ uptake of 8.37 ± 1.37 pg/cell (Figure 3B). The 30 nm AgNPs displayed the lowest recorded AgNP concentrations of the three NP sizes, with just 0.27 ± 0.07 pg/cell following exposure to 2.5 µg/mL, while the medium and high concentrations showed statistically significant differences compared to naive cells (** *p* < 0.01). On the other hand, the 100 nm AgNPs presented higher uptake values than the 30 nm particles, but less than that of the 10 nm AgNPs. The 2.5 µg/mL exposure of the 100 nm AgNPs resulted in uptake of 0.067 ± 0.05 pg/cell, while the medium and high concentrations were statistically different compared to the naive cells (* *p* < 0.05); the highest recorded value was 5.92 ± 0.20 pg/cell following exposure at 10 μg/mL (Figure 3B).

Differences in the total Ag^+^ between timepoints can be related to different factors, the most likely being NP dissolution and consequent internalisation of ionic Ag, especially at high NP concentrations and for the 10 nm particles, whereas the large NPs may suffer from agglomeration, influencing their uptake at longer incubation times. The drop in the total Ag^+^ uptake for the 30 nm could be related to the reduced availability of Ag^+^ ions in the cell medium due to lower dissolution rates, as demonstrated in a previous study [27] and further discussed in the next section. The full results for cellular internalisation (total Ag) can be found in Supplementary Materials Table S3 in the Supplementary Materials.

### 3.4. Inhibition of the Cellular Uptake Pathways Demonstrated by Confocal Microscopy

Different pharmacological inhibitors can be used to block the three main endocytic pathways, including genistein, which is an inhibitor of the tyrosine kinases involved in caveolae-mediated endocytosis; chlorpromazine, which inhibits clathrin disassembly and its receptor from the cell membrane during clathrin-mediated endocytosis; and wortmannin, which inhibits membrane phospholipids such as phosphoinositide-3-kinase (PI3K) and is closely interrelated with the activation of macropinocytosis [8,9]. The effectiveness of pharmacological inhibitors in blocking the desired pathway was tested with positive controls for the specific pathways, including transferrin (Tf), which enters cells through clathrin-dependent endocytosis; cholera toxin beta subunit (hereafter called cholera toxin b), linked to caveolae dependent endocytosis; and dextran, a probe for the macropinocytosis pathway [6,8,9,11]. Additionally, to understand the effects of inhibitors on cells, the cell viability of the tested inhibitors at the selected concentrations were evaluated by lactate dehydrogenase (LDH) assay, in order to find the lowest concentration at which the inhibitor is still active. The methodology used for the LDH assay can be found in Section 4.1 of the Supplementary Materials.

**Figure 4:**
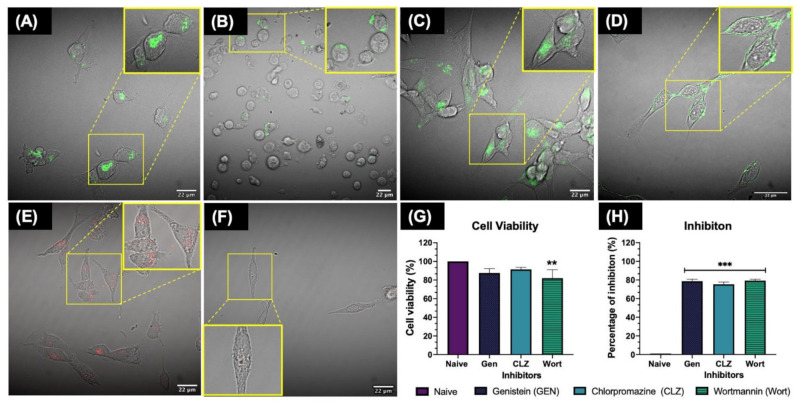
Inhibition of the cellular uptake pathways. (**A**) Uptake of cholera toxin b (control); (**B**) Almost complete reduction of cholera toxin b uptake following inhibition of the caveolae pathway with Genistein at 100 µg/mL for 20 min. (**C**) Uptake of transferrin (control); (**D**) Reduction of transferrin uptake by the clathrin-mediated pathway after treatment with 10 µg/mL chlorpromazine for 30 min; (**E**) Uptake of dextran (control); (**F**) Almost complete reduction in uptake of dextran by the macropinocytosis and phagocytosis pathways after treatment with 10 µg/mL wortmannin for 10 min. All the controls were incubated for 2 h after the addition of the chemical inhibitor. (**G**) Cell viability (%) assessed by LDH assay after treatment with the selected inhibitor concentrations and incubation period. (**H**) The percentage of inhibition calculated for the inhibitor images against their controls. Three individual replicates were performed for each experimental condition and analysis. Images were taken with a 60× objective lens using a NIKON A1R 808 microscope (Nikon, Tokyo, Japan). Data with asterisks (*) indicate a statistically significant difference (** *p* < 0.01 and *** *p* < 0.001) compared to the naive cells.

Confocal microscopy demonstrated that the uptake of 1 μg/mL of cholera toxin b (Figure 4A) was successfully inhibited after treatment with (100 μg/mL) genistein for 20 min (inhibition of the caveolae pathway) (Figure 4B). Genistein treatments presented visible morphological effects, (for example, cells became rounded compared to the flat and elongated morphology present in the untreated controls); however, the evaluated cytotoxicity showed a cell viability of 87.56 ± 4.67% after 20 min, compared to the control with 100% of viability (Figure 4G). The results for chlorpromazine (10 μg/mL) displayed less cytotoxicity, with 91.41 ± 2.31% cell viability after 30 min of incubation. Confocal images demonstrated inhibition of the receptor via the reduced uptake of transferrin (500 μg/mL), a ligand exclusively internalised via the clathrin-mediated endocytosis pathway, as shown in Figure 4C,D.

The wortmannin results displayed higher reduction of cell viability, with 82.04 ± 9.01% viability after 10 min of incubation; however, the concentration of 10 μg/mL proved to be the lowest that can be used to achieve an inhibitory effect of the macropinocytosis pathway as shown in Figure 4F, where some small amount of uptake was still observed compared to the control (dextran) (Figure 4E,F). Most of the inhibitors displayed a reduction in cell viability <20%, proving that the selected concentrations were suitable to induce around 80% of inhibition for all the tested inhibitors (Figure 4H) and for the specific endocytic pathway. Other studies in human, mouse, and fish cell lines agree with these findings, highlighting cytotoxicity and reduction of the cell viability after exposure to pharmaceutical inhibitors [8,13,14,36]. The full results can be found in Supplementary Materials Table S4 in the Supplementary Materials.

### 3.5. Quantification of the Uptake of Ag^+^ during Exposure to AgNPs Following Inhibition of the Cellular Uptake Pathways of ZF4 Cells

**Figure 5:**
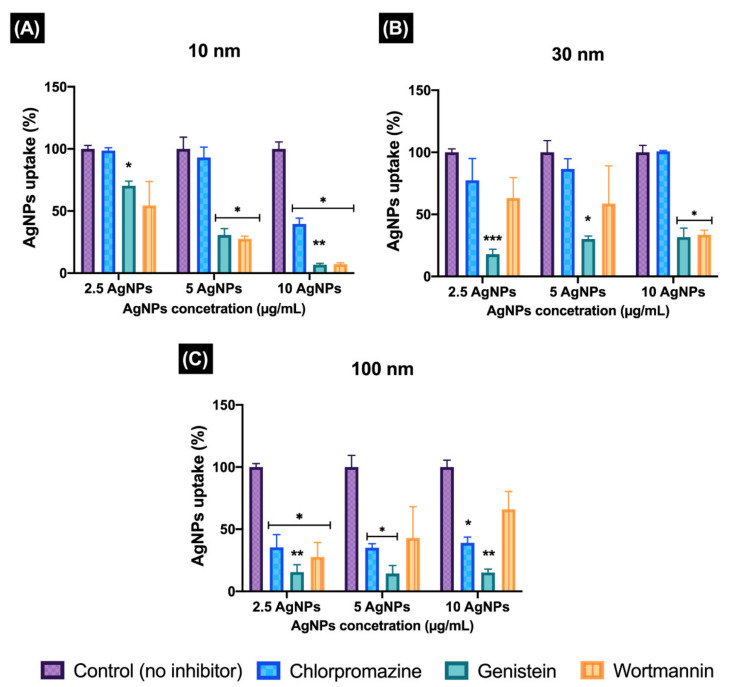
Quantification of the inhibition of the cellular uptake pathways determined as % AgNPs internalised. ZF4 cells were treated with either (**A**) 100 µg/mL of genistein for 20 min, (**B**) 10 µg/mL of chlorpromazine for 30 min, or (**C**) 10 µg/mL of wortmannin for 10 min. After treatment with the chemical inhibitor, cells were treated with 2.5, 5, and 10 µg/mL of different AgNP sizes (10, 30, and 100 nm) for 2 h. ICP–MS results for total Ag were normalised to percentage (%) against their untreated control (no inhibitor) for each AgNP concentration and size. Results represent the mean of three individual replicates and their standard deviation. Data with asterisks (*) indicate a statistically significant difference of the inhibitor treatments (* *p* < 0.05, ** *p* < 0.01, and *** *p* < 0.001) compared to the naive cells (non-inhibition) at each time point. All bars under the brackets are included within the asterisk above.

Once the inhibitory concentrations proved to be effective at inhibiting the desired pathway, the internalisation of AgNPs (total Ag^+^) was quantified by ICP–MS, following inhibition of each pathway. The AgNPs uptake was measured only at 2 h, as blocking of one uptake pathway can result in activation of other endocytic mechanisms, which may confound the results [8,14]. The results for the levels of inhibition in the uptake of AgNPs are presented as a percentage (%) with respect to the uptake obtained by ICP–MS in normal cells exposed to AgNPs under the same conditions and without any inhibitor treatment (Figure 5). The normalised results show different patterns across the different treatments with the inhibitors, AgNPs sizes, and concentrations used. The 10 nm AgNPs showed a clear uptake-inhibition response for all chemical treatments and uptake pathways assessed. The results for the CLZ treatments showed no significant differences for the low and medium concentrations (2.5, and 5 μg/mL) compared to the naive (100%), both with a percentage uptake of around >93%; however, the 10 μg/mL showed a significant difference (* *p* < 0.05), with a low percentage uptake of 39.54 ± 4.67%. Genistein treatments displayed significant differences (* *p* < 0.05, ** *p* < 0.01) compared to the uptake of the control; in particular, the medium and high concentrations showed a reduction in uptake of the 10 nm AgNPs with 30.71 ± 5.13% and 6.73 ± 1.12% uptake, respectively, relative to the non-inhibited exposures (100%). The wortmannin treatments displayed a major reduction of uptake at the low and medium concentrations (54.48 ± 19.20 and 27.61 ± 2.12%, respectively), followed by a statistically significant decrease (* *p* < 0.05) at the highest concentration (7.27 ± 1.05%), compared to the uninhibited control.

The 30 nm AgNPs showed an inverse trend for CLZ treatments, with a greater inhibition response at the lowest AgNP (2.5 mg/mL) concentration (77.38 ± 17.57), followed by 86.53 ± 8.32 for the 5 μg/mL, and no inhibition at all for the 10 μg/mL (100%), suggesting that the clathrin-mediated pathway is not the preferred pathway in ZF4 cells at medium AgNP sizes. Genistein treatments showed significant differences for all AgNP concentrations (* *p* < 0.05 and *** *p* < 0.001), with similar percentages of inhibition for the high and medium AgNPs concentrations (30%), but with a noticeable decrease at the lowest concentration (2.5 μg/mL), with an uptake of 17.90 ± 4.03% of the control. On the other hand, wortmannin treatments showed a different pattern for the 30 nm AgNPs, with a clear increasing concentration-inhibition response, with uptake reduced to 63.16 ± 16.47% relative to the untreated control at the lowest AgNP concentration, and to 33.67 ± 3.60% for the 10 μg/mL.

Treatments with the 100 nm AgNPs and CLZ displayed similar results for the three AgNPs concentrations, with reduction in uptake percentages (to around 30%) for all concentrations. Genistein treatments resulted in statistically significant differences in uptake of the 100 nm AgNPs compared to the naive (** *p* < 0.01) cells at the low and high concentrations, suggesting that the caveolae pathway is one of the major routes of internalisation for this NP size (100 nm). The results for wortmannin showed that inhibition was most effective at the low exposure concentrations, but less effective at higher 100 nm AgNP doses, showing the lowest uptake at 2.5 μg/mL (27.70 ± 11.79), followed by 42.99 ± 25.20% uptake for the 5 μg/mL, and finally 66.06 ± 14.31% at 10 μg/mL 100 nm AgNPs relative to the naive cell uptake.

Overall, the results suggest that the uptake of the 10 nm AgNPs is dominated by macropinocytosis and the caveolae pathway. The 30 nm size showed most inhibition in the genistein treatments, suggesting that the caveolae pathway is responsible for the uptake at this size. The 100 nm size similarly showed caveolae pathway inactivation, followed by clathrin-mediated, and lastly the macropinocytosis pathway, but indicated that all pathways can be activated for NP uptake at this size. A summary of the results can be found in Supplementary Materials Table S4 in the Supplementary Materials.

### 3.6. Early Endosomes Induction (EEI)

Early endosomes (EE) are the first endocytic compartment to sort internalised cargo such as lipids, proteins, and extracellular molecules contained within the endocytic vesicles (from the cell membrane) to different intracellular destinations [37]. Hence, to gain further insights about the internalisation of the AgNPs in ZF4 cells, the EE induction (EEI) was evaluated by labelling the EE with red fluorescent protein after 2 and 24 h of incubation. For this, intensity values were normalised and plotted as percentage (%) of induction with respect to naive, which was considered as 0% or baseline (see Materials and Methods for calculations), for easier interpretation.

**Figure 6:**
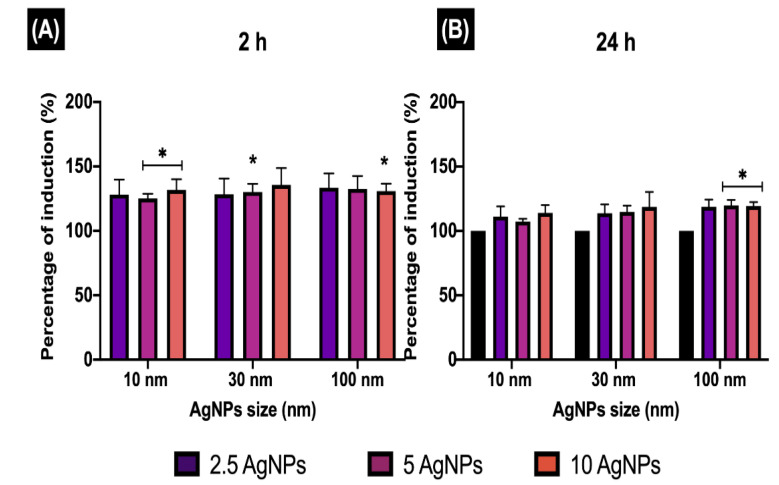
Early endosome induction. The percentage of EE induction (EEI) in ZF4 cells treated with 2.5, 5, and 10 µg/mL of three different AgNP sizes (10, 30, and 100 nm) after (**A**) 2 and (**B**) 24 h. The intensity results were normalised to percentage (%) relative to the untreated cells (naive) (see Materials and Methods Section 2.6 for calculation). Data represent the mean of three individual replicates and above the bars the standard deviation (Mean ± SD). Data with asterisks (*) indicate a statistical difference (* *p* < 0.05) between the treatments compared to the untreated control (naive) at the specific timepoint.

The normalised results (Figure 6) displayed higher percentages (%) of induction after 2 h (>30%) than after 24 h for all NP sizes and concentrations. After 2 h, the 10 nm AgNPs displayed the highest % EEI at the highest exposure concentration 10 µg/mL (131.7 ± 8.35%). Statistically significant differences (* *p* < 0.05) where found for the medium and high concentrations (5 and 10 µg/mL) when compared to the untreated control (naive). After 24 h, the 10 nm AgNPs showed the lowest % EEI at the low and medium concentrations, with 111.13 ± 7.91% for 2.5 µg/mL and 107.18 ± 2.22% for 5 µg/mL, whereas the 10 µg/mL showed a slightly higher value, with 114.00 ± 4.91%.

On the other hand, the 30 nm size displayed a concentration-dependant trend after 2 h: the results for 2.5 and 5 µg/mL showed values between 128% and 130%, whereas the 10 µg/mL result in % EEI of 135.61 ± 13.06% (Figure 6). After 24 h, the % EEI was lower for the high and medium concentrations (compared to 2 h), with values ranging from 114 to 118%, whereas the lowest concentration (2.5 µg/mL) displayed a similar %EEI (131.7 ± 114.7) as that recorded at 2 h.

The increased EEI response at shorter exposure times (2 h) was also evident for the 100 nm AgNPs, although, this size displayed an inverse concentration-dependant trend, with values between 130% and 133%. After 24 h, values were similar at all exposure concentrations, around 118–119%, showing statistical differences (* *p* < 0.05) at 5 and 10 µg/mL compared to naïve cells.

Overall, the results (Figure 6) demonstrated that the EEI is closely linked to the size of the NPs, with higher EEI percentages for the larger size (100 nm), compared to the other NP sizes (10 and 30 nm) at both time points. Exposure time also played a key role in the EEI, with higher trafficking and induction of EE during the first two hours, compared to 24 h, which showed a much lower % of EEI for all the NP sizes.

### 3.7. Autophagy Response

Autophagy is a lysosome-based degradative pathway that helps maintain intracellular homeostasis and degradation of molecules [10]. Under normal conditions, cells maintain low levels of autophagosome formation, as they rely on other uptake methods to eliminate external matter; thus, to further investigate the induction of autophagy in ZF4 cells during the exposure of AgNPs, an autophagosome maker was labelled, and its induction was assessed.

**Figure 7:**
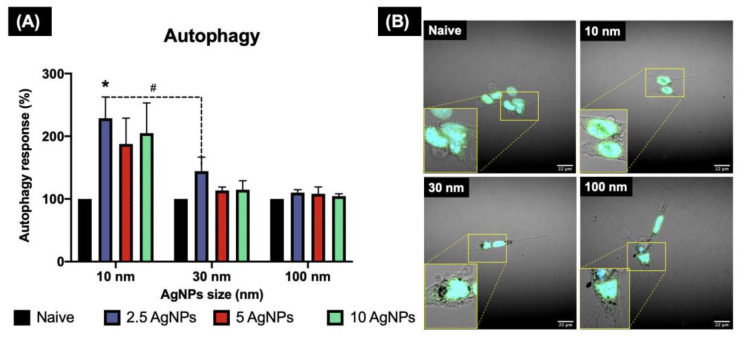
Autophagy induction in ZF4 cells exposed to AgNPs as determined by confocal microscopy. ZF4 Cells were treated with 2.5, 5, and 10 µg/mL AgNPs of three different sized AgNPs (10, 30, and 100 nm) for 24 h. Images of the cells with the nucleus (blue) and autophagosome staining (green) were taken at 60× with a NIKON A1R 808 series microscope (Nikon, Tokyo, Japan). A close-up of the labelled cells is marked with yellow lines. The intensity of the labelled autophagosomes in ZF4 cells was recorded by FIJI. (**A**) Results of three individual replicates are expressed as mean and standard deviation (Mean ± SD). A statistical comparison between all the treatments was performed. Data with asterisks (*) indicate a statistical difference (* *p* < 0.05) between the treatments compared to the untreated control (naive). The symbol above the bars (#) represents a statistically significant difference (^#^ *p* < 0.05) between the marked treatments. (**B**) Cells treated with 10 µg/mL of different AgNPs sizes and the control. Images for the remaining AgNP concentrations and sizes can be found in the Supplementary Materials (Figure S4).

The results for the autophagy induction displayed the highest percentages (%) for the smallest NP (10 nm), compared to the other NPs sizes (Figure 7). Here, the low and high concentrations showed induction values above 200%, whereas the medium concentration (5 µg/mL) displayed 187.62 ± 41.27%. A statistically significant difference (*p* < 0.05) for the 2.5 µ/mL against naive was found for the 10nm AgNPs. Similarly, a multiple comparison between the NPs sizes and concentrations showed a statistically significant difference (*p* < 0.05) between 10 and 30 nm for the lowest concentration (2.5 µg/mL). The 30 nm AgNPs showed the highest % autophagy induction at the lowest AgNP concentration (144.19 ± 22.49%) compared to the medium and high concentrations (113.60 ± 5.41 and 114.73 ± 14.24%, respectively). The 100 nm AgNPs presented the lowest recorded % of autophagy induction of the three NP sizes, and showed a similar trend as the 30 nm, with a higher % induction at the lowest concentration (110.15 ± 4.56%), compared to the medium and high concentrations, with 108.15 ± 10.99 for 5 µg/mL and 104.55 ± 3.64% for the 10 µg/mL. Normalised data and the remaining confocal images can be found in Supplementary Materials Table S6 and Figure S4 in the Supplementary Materials.

### 3.8. Protein Corona Isolation and Analysis of Proteins by PAGE

The isolation of the protein coronas formed on the different AgNP sizes after exposure at different mass concentrations for different times revealed that the corona formation process can be strongly mediated by these factors. Bands in the gels were highly visible for the smaller NPs (10 nm) after 24 h of exposure compared to 2 h in CCM. For example, after 2 h, gel bands for all three AgNP sizes and concentrations were similar, showing a single line between 72 and 57 kDa (Supplementary Materials Figure S5A), whereas after 24 h, the 10 nm AgNPs at the highest concentration displayed a larger number of proteins across the band (Supplementary Materials Figure S5C). This suggests that the concentration of the NPs can influence the composition of the coronas, perhaps due to the secretion of certain proteins by the cells as a result of the induction of endocytic pathways, increasing the availability of proteins in the cell medium as well as the dynamic behaviour of the NP-protein binding process. To further understand the role of the secreted proteins, an initial experiment to record changes in the protein concentrations in the ZF4 cell medium during AgNP exposure was performed by recovering particles that had not yet been internalised. The results demonstrated that the total protein concentration (in suspension) increased over time for all the NP sizes and concentrations, with approximately a 5–10% increase of the total protein concentration in the medium after 24 h, compared to the untreated control, which remained with a constant protein concentration after 24 h (Supplementary Materials, Section S7.1, Supplementary Materials Figure S6). However, further studies are necessary to confirm the mechanism triggered by the NPs as reflected in the increased protein expression.

## 4. Discussion

The physicochemical properties of NPs can strongly influence their toxicity in biological systems. However, these characteristics may substantially change upon introduction of the NPs into a complex biological environment, leading to different outcomes [11,15,16]. In this study, we demonstrated that the characterisation in ultra-pure water (UPW) and complete culture media (CCM) displayed different results. The addition of proteins strongly affected the NPs’ characteristics, creating a new biological identity (protein corona), that was reflected in the hydrodynamic size and polydispersity index (PDI), in CCM that was more than double those in water, influencing the NPs’ interactions with the biological system [11,38,39]. Similarly, the protein components in the biological media also affected the surface charge, as seen in the zeta potential results in CCM, which became lower than in water, around −9 mV (for all NPs), as consequence of the serum protein adsorption onto the NPs [8,40]. The absorption spectra measured by UV–Vis were also strongly influenced by the biological medium; in the complex media, the samples exhibited significant scattering increase (compared to in UPW) and a second peak, revealing the presence of proteins in the sample. These results can be related to the fact that a considerable fraction of transmitted light does not reach the UV–Vis detector due to the complex nature of the cell medium, recording a higher absorption for these samples [41].

The intracellular uptake and fate of the NPs can also be influenced by their physicochemical properties, cell type, and constituents in the complex medium, such as proteins, that can modulate the cellular uptake, for example, by enabling the NPs to engage cellular receptors following formation of the protein corona [42,43,44]. In the present study, we found a strong dependence of the AgNP protein corona composition on the time of exposure and the size of the NPs. Isolation of the protein corona by SDS-PAGE illustrated a clear difference in the protein corona identity and evolution over time for the two biological incubation periods. After 2 h, the gel bands showed a single line between 72 and 57 kDa (Supplementary Materials Figure S3), which may be related to common proteins found in FBS, such as serum albumin (66 kDa), as well as complement proteins such as immunoglobulin, and apolipoproteins [45,46]. Similarly, other serum proteins such as α_1_-antitrypsin (52 kDa) and keratin type ii cytoskeletal (57–70 kDa) have also been found as part of the protein corona of PVP-AgNPs in CCM (10% FBS) in a different study [45]. The SDS-PAGE gel also revealed that after 24 h (Supplementary Materials Figure S3), the smaller AgNPs (10 nm) presented a number of highly visible protein lines along the length of the band for the medium and high AgNP concentrations, showing a clear separation of the protein sizes, ranging between 250 and 258 kDa. This can be related to other serum- related proteins such as Apolipoprotein A-I (28 kDa) and Apolipoprotein A-II (17.4 kDa) [45], as well as specific ZF4 cellular proteins secreted by the cells in response to damage induced by the AgNPs, including p65 transcription factor (51.1 kDa), retinoic acid receptor RXR-gamma-A, and max-interacting protein 1 (27.6 kDa), proteins that can be found in the nucleus and are related to DNA regulation and binding transcription activity; and the swelling-dependent chloride channel protein (27.4 kDa), which is located in the cytoplasm, nucleus, and plasma membrane of ZF4 cells; and ataxin-3 Fragment (34.5 kDa), involved in protein de-ubiquitination and located in the nucleus [47]. A full list of the identified proteins can be found in Supplementary Materials Table S7 in the Supplementary Materials.

The differences between the NPs sizes and their protein composition can perhaps be related to the curvature of the NPs, and to the fact that the 10 nm solution contains a much larger number of particles (NPs/mL) and a much larger surface area for protein adsorption compared to the larger AgNPs at constant mass [27]. In this regard, small NPs have a higher total Surface Area (SA), which may enable more protein binding compared to the larger sizes, as demonstrated in a previous publication [27]. Calculations were performed to obtain the total SA for the NPs based on their hydrodynamic size, demonstrating that the smaller NPs have a higher SA (6.22 × 10^−5^ m^2^/g), compared to the 30 nm (5.78 × 10^−5^ m^2^/g) and the 100 nm size (3.67 × 10^−5^ m^2^/g), which was 1.69× smaller than the 10 nm AgNPs (results for highest AgNP concentration). This confirms that that smaller NPs may have a higher likelihood of binding proteins due to their large SA, as suggested by other authors [3,45,48]. The SA results for other concentrations can be found in Quevedo et al., 2021 [27].

Another aspect to consider is the type of proteins in the complex environment; for example, albumin and fibrinogen are proteins that will bind and dominate the particle surface due to higher abundance, especially at short exposure times. These proteins may eventually be displaced by proteins with higher affinity and slower kinetics, increasing the protein diversity of the protein corona [44]. For example, certain proteins secreted by ZF4 cells could have a higher affinity for the AgNPs compared to the serum proteins, potentially increasing the competition of proteins and displacement of weakly bound proteins; however, further studies are necessary to fully confirm the role of the secreted proteins in driving AgNP uptake by ZF4 cells [39,44].

On the other hand, the uptake of AgNPs may also be strongly related to the size of the NPs and the time of exposure, as suggested by other authors [49,50]. Our results for the total Ag in cells demonstrated that after 2 h, the 10 nm AgNPs displayed an overall average uptake of 10% (calculated as the average of the three concentrations) of the available dose, whereas the larger AgNP sizes (30 and 100 nm) displayed an uptake efficiency per cell between 0.3% and 3% of the exposure dose for all concentrations. After 24 h, the uptake for all the sizes greatly increased, with the 10 nm AgNPs showing the highest percentages of uptake, displaying almost 90% uptake for the highest concentration (10 µg/mL), followed by the 100 nm size, with an overall average of 40%, and lastly the 30 nm, with an average uptake of 19%. Interestingly, the larger AgNP size (100 nm) displayed higher percentages of uptake compared to the medium size (30 nm). It has been suggested that there might be an optimal particle size for active uptake; for example, in human cell lines exposed to different AgNPs sizes, the 50 and 100 nm had higher uptake efficiencies compared to the smaller size (20 nm) [49,50]. Similarly, Qiang et al., 2020 suggested the smaller sizes up to 20 nm are more likely to be internalised than larger sizes, providing evidence for enhanced uptake and toxicity for smaller sizes (4 nm) in zebrafish embryos [51]. Another study demonstrated that the uptake efficiency of 5 and 100 nm AgNPs after 24 h was higher (58 and 63% respectively) compared to other sizes (20 nm and 50 nm) [49], supporting our findings. It is important to mention that the detected total Ag^+^ intracellular concentrations can also be linked to the dissolution of the NPs; for example, in a previous study, the 10 and 100 nm presented higher dissolution and uptake values compared to the 30 nm AgNPs in CCM [27]. This suggests that the high total Ag^+^ intracellular concentrations detected by the 10 nm can be linked to a major abundance of dissolved ions in the media due to the high rates of dissolution, whereas the 100 nm results can be a combination of dissolution and sedimentation, which may increase their contact with the cells, and therefore their uptake amount [27]. Thus, a mixture of NPs and ionic uptake is likely to occur in ZF4 cells.

Analysis of the TEM images demonstrated the internalisation of AgNPs as well as the formation of vesicles as part of the endocytic process is activated by ZF4 cells, to deal with exposure to the NPs. Analysis of the internalised NP size indicated a reduction in their initial size, as well as visible loss of density or fragmentation once inside the vesicles for all the NPs, most evident for the 10 nm AgNPs (see Figure 2C). The TEM images showed that the small and medium AgNPs (10 and 30 nm) were located close to the nucleus (see Figure 2A,D), as magnification of the images revealed NPs in the nucleus area. This can be related to the easier cellular internalisation of the NPs due to their small size, as well as to the fact that NPs may be able to escape from the vesicles [52,53]. This process has been described as the enhanced Trojan horse effect, which refers to the cellular internalisation of metal NPs via an active processes, resulting in an enhanced release of toxic ions such as Ag^+^ as a result of cation-induced lysosomal damage or dysfunction [54].

Other studies agree with these findings; for example, Berry et al., (2007) demonstrated that small gold nanoparticles (5 nm) were visible in the nucleus of human fibroblasts, whereas larger particles (>30 nm) were observed mainly in the cytoplasm, suggesting that the fate of the internalised NPs can also be controlled by the dimensions of the organelle membranes, such as the nuclear pores in the nucleus [55]. Interestingly, the TEM images for 100 nm showed a larger number of NPs surrounding the cell membrane, with no visible signs of agglomeration. A study by Greulich et al., (2011) demonstrated that large AgNPs (80 nm) were visible in the cytoplasm, and were able to be internalised in hMSC cells as nanoparticulate material and agglomerate once inside the perinuclear region, which is connected to the endo-lysosomal cell compartment [56]. The NPs’ degradation and their reduction in diameter can also be related to their dissolution (as previously discussed), as well as the lysosomal degradation by hydrolases, which can digest proteins, nucleic acids, lipids, and extracellular agents, as described by other studies [54,57].

Once the NPs are engulfed by the cell membrane during any endocytic process, the formation of EE is rapidly activated to sort the internalised cargo to different intracellular destinations [37]. The internalised cargo remains for only a few minutes in EE (between 5 and 10 min); then, the EE progressively acidify and mature to late endosomes [37]. Interestingly, the results for the EE induction (EEI) revealed higher NP trafficking during the first 2 h for all NP concentrations and sizes, compared to longer exposure times (24 h). The internalisation and detection of AgNPs was confirmed in ZF4, demonstrating the ZF4 cells activate endocytosis processes to deal with the NP exposure. The results for the uptake pathways induced by the AgNPs demonstrated that different endocytotic pathways may take place simultaneously, involving complex NP-cell interactions in their uptake by ZF4 cells, as suggested by other authors [24,58]. Size and mass concentration also proved to affect the uptake pathway during the assessment with pharmaceutical inhibitors. The results for the inhibition of the endocytosis pathways showed that treatment with chlorpromazine has no effect on the uptake of 10 and 30 nm AgNPs; therefore, the clathrin pathway was not activated for most of the concentrations, except at the 10 µg/mL for the 10 nm size. Here, the caveolae-mediated and macropinocytosis pathways were mainly responsible for the uptake of the 10 nm AgNPs, showing similar percentages of uptake by both pathways. These results agree with a study by Gunduz et al., (2017), who demonstrated that 12 nm AuNPs predominant entered HUVECs via macropinocytosis [59]. Similarly, NPs could also be internalised due to their small size during the engulfment of large volumes of the extracellular medium, which allows the internalisation of fluid-phase nutrients such as proteins and ATP, and sampling of the environment for foreign agents [9]. In addition, it has been suggested that cells may internalise secreted vesicles, called exosomes, through macropinocytosis, which can be related to the fact that small NPs are easily internalised (as seen in the uptake data); so perhaps cells are coping with the exposure by encapsulating the NPs inside vesicles, recycling these vesicles to the cell membrane, and then internalising these vesicles again via macropinocytosis [9,60].

On the other hand, the 30 nm AgNPs displayed inhibition of the caveolae pathway at all AgNPs concentrations tested; similar results were displayed by the 100 nm size for this pathway, which also showed inhibition of the other evaluated pathways. These results suggest that the 30 and 100 nm uptake are linked to the strong participation of lipid raft-associated receptors during their internalisation [8]. These results are in agreement with other authors who demonstrated a clear inhibitory effect of genistein treatments on the uptake by human cells of large NPs such as 200 nm PS-COOH NPs and 500 nm latex microspheres, suggesting that caveolae flask-shaped invaginations can internalise larger particles compared to clathrin-coated pits, due to the restricted triskeletal structure of clathrin [8,61]. It is important to mention that the NP uptake was measured only at short exposure times (2 h), as it has been reported that the inhibition of one uptake pathway can result in the activation of other endocytic uptake pathways [8,14]. When selecting a specific inhibitor for the desired endocytosis pathway, it is important to take into account the effective inhibition as well as cytotoxicity of the pharmacological inhibitors, before their co-incubation with the NPs, as severe disruption (in terms of toxicity) may inactivate or activate other molecular processes as part of the cell’s defence mechanism, which may lead to different outcomes [8].

Autophagy as endocytosis is involved in the formation of membrane vesicles for the degradation and recycling of intracellular and extracellular components; thus, it could be considered another (non-endocytotic) pathway by which cells could internalise NPs [62]. It has been suggested that both pathways are interconnected at different stages during the formation, fusion, and trafficking of the vesicles, having a common endpoint at the lysosomes, where the internalised cargo is degraded, and finally eliminated by exocytosis [62]. The results for the induction of autophagy showed higher induction (%) for the small NPs, whereas the medium and large NPs showed less autophagy levels (<1%). This suggests that autophagy could be directly related to the size of the NPs and the level of stress the cells are under [10]. In addition, autophagy could potentially be activated during macropinocytosis and caveolae-mediated endocytosis, which were the pathways that showed the highest inhibition in this study. For example, a study by Zhang et al., (2017) has demonstrated that polymeric NPs (PLGA) were also internalised by the autophagy pathway, as NPs were observed in autophagosomes in human cells, suggesting a close link between autophagy and endocytosis [63]. The increased autophagy response in our study could also be linked to the cytotoxicity of the AgNPs in ZF4 cells, which has been previously demonstrated [27]. Autophagy is primarily described as a self-protective mechanism; thus, excessive levels of autophagy could be linked to undergoing stress caused by damaged organelles, which result in an alkalising effect on the endosomal system, reducing the functionality of the formed endosomes, causing induction of autophagy to remove the extracellular components and damaged organelles to cope with the NPs [53].

The cellular internalisation methods assessed in this study provide further insights about the uptake and fate of NPs in cellular models. Besides, fish cell lines have been further described as potential in vitro models for chemical and water testing, including ZFL and ZF4 cell lines [23,64]. In this regard, the assessed toxicological responses in ZF4 cells can support the transition towards the replacement, reduction, and refinement of animals in experimentation, by providing evidence to encourage the application of the 3Rs in regulatory ecotoxicity testing [29,64]. Similarly, ZF4 cells can work well as a potential model to support the transition to more sustainable and alternative toxicity testing, and for facilitating the acquisition of data for the AOP framework for a 21st century toxicological assessment.

## 5. Conclusions

The present study demonstrated the internalisation mechanisms of three representative AgNP sizes (10, 30, and 100 nm) in embryonic zebrafish cells (ZF4). The inhibition of the caveolae, clathrin, and macropinocytosis endocytic pathways by pharmaceutical inhibitors (genistein, chlorpromazine, and wortmannin, respectively) confirmed that ZF4 cells’ uptake occurred via different endocytosis mechanisms, depending on the particle size. For example, the results revealed that uptake of the 10 nm size was mainly via macropinocytosis, whereas the uptake of the 30 and 100 nm sizes was mediated via the caveolae-mediated pathway.

In addition, we demonstrated that the AgNP mass concentration, size, and time of exposure of the three different AgNP sizes were closely linked to their intracellular fate, as displayed in the TEM images indicating the formation and disruption of early endosomes, and the activation of the endocytic uptake pathways, such as clathrin, caveolae macropinocytosis, and autophagy as a non-endocytic process. The presence of NPs inside the cells, including in the cytoplasm and close to the nucleus, was confirmed by TEM and correlated with the total Ag content as determined by ICP–MS, although the presence of intact 10 nm AgNPs was challenging to confirm, as the particles underwent some dissolution following internalisation.

Finally, the results presented in this study provide further insights about the cellular uptake mechanisms in ZF4 cells exposed to NPs, highlighting the complexity and interplay between induced intracellular mechanisms and NPs. The confirmation of existence and availability of the well-known endocytotic pathways in the ZF4 cells is an important step towards widespread adoption of this alternative vertebrate model for high-throughput in vitro toxicity testing, supporting the 3Rs initiative for the refinement, reduction, and replacement of animals in experimentation, for safer and sustainable nanotoxicological assessment.

## Data Availability

All data are included in the Supplementary Materials.

## Supplementary Materials

The following are available online at https://www.mdpi.com/2079-4991/11/10/2699/s1, Figure S1. Characterisation of the AgNPs by TEM. Table S1. Characterisation of the AgNPs. Figure S2. UV–vis images of the AgNPs. Figure S3. Analysis of intracellular AgNPs and vesicles. Table S2. Intracellular AgNP size determined by TEM. Table S3. Total Ag+ concentration per cell (pg/cell) determined by ICP–MS. Table S4. Inhibition of uptake pathways and resulting changes in AgNP uptake and internalisation. Table S5. Early endosome induction. Table S6. Autophagy results. Figure S4. Images of autophagy induction. Table S7. Analysis of the corona compositions. Figure S5. Coomassie blue staining of PAGE gels for isolated protein corona. Figure S6. Proteins secreted during AgNPs exposure in medium containing 10% FBS.
