# Correction: Quevedo et al. Mechanisms of Silver Nanoparticle Uptake by Embryonic Zebrafish Cells. *Nanomaterials* 2021, *11*, 2699

**DOI:** 10.3390/nano12020225

**Published:** 2022-01-11

**Authors:** Ana C. Quevedo, Laura-Jayne A. Ellis, Iseult Lynch, Eugenia Valsami-Jones

**Affiliations:** School of Geography, Earth and Environmental Sciences, University of Birmingham, Edgbaston, Birmingham B15 2TT, UK; L.A.Ellis@bham.ac.uk (L.-J.A.E.); E.ValsamiJones@bham.ac.uk (E.V.-J.)

**Error in Figure/Table**

In the original publication [1], two figures unrelated to the current manuscript were inserted during the copyedit stage. Thus, Figure 6 and Figure 7 are incorrect. The Editorial office apologizes for this error and for any inconvenience caused and the authors confirm that the scientific conclusions are unaffected. The original publication has also been updated with the correct versions of Figure 6 and Figure 7, which are included here also.

**Addition of an Author**

**Laura-Jayne A. Ellis** was not included as an author in the original publication. The corrected Author Contributions Statement appears here. The authors apologize for any inconvenience caused and state that the scientific conclusions are unaffected. The original publication has also been updated.

**New Author Contribution statement:**

**Author Contributions:** Formal analysis, A.C.Q.; Methodology, A.C.Q., L.-J.A.E.; Supervision, I.L. and E.V.-J.; Writing—original draft, A.C.Q.; Writing—review & editing, I.L. and E.V.-J. All authors have read and agreed to the published version of the manuscript.

## Figures and Tables

**Figure 6:**
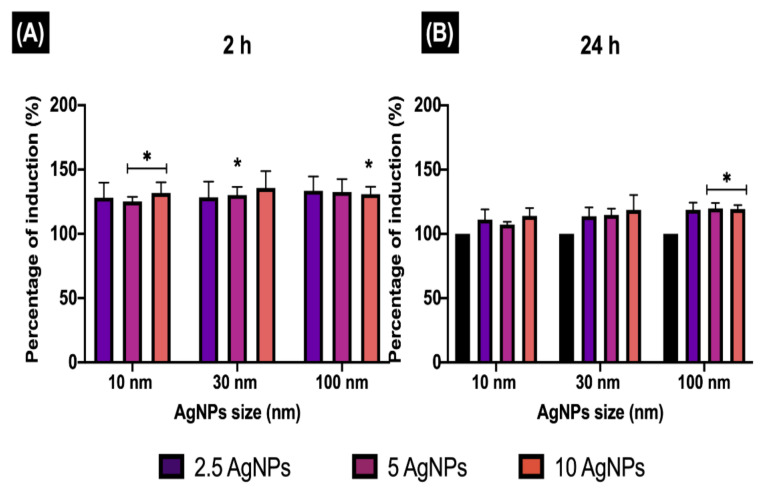
Early endosome induction. The percentage of EE induction (EEI) in ZF4 cells treated with 2.5, 5, and 10 µg/mL of three different AgNP sizes (10, 30, and 100 nm) after (**A**) 2 and (**B**) 24 h. The intensity results were normalised to percentage (%) relative to the untreated cells (naive) (see Materials and Methods Section 2.6 for calculation). Data represent the mean of three individual replicates and above the bars the standard deviation (Mean ± SD). Data with asterisks (*) indicate a statistical difference (* *p* < 0.05) between the treatments compared to the untreated control (naive) at the specific timepoint.

**Figure 7:**
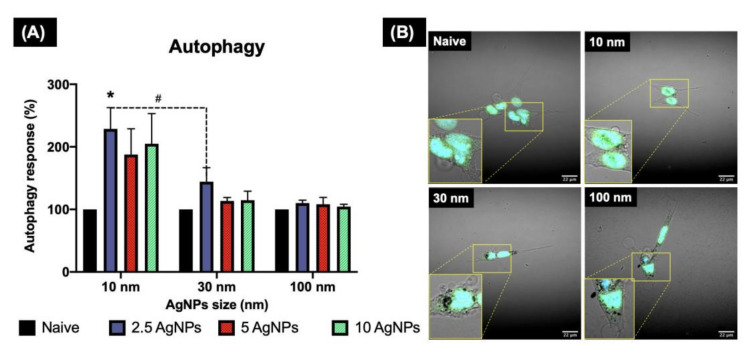
Autophagy induction in ZF4 cells exposed to AgNPs as determined by confocal microscopy. ZF4 Cells were treated with 2.5, 5, and 10 µg/mL AgNPs of three different sized AgNPs (10, 30, and 100 nm) for 24 h. Images of the cells with the nucleus (blue) and autophagosome staining (green) were taken at 60× with a NIKON A1R 808 series microscope (Nikon, Tokyo, Japan). A close-up of the labelled cells is marked with yellow lines. The intensity of the labelled autophagosomes in ZF4 cells was recorded by FIJI. (**A**) Results of three individual replicates are expressed as mean and standard deviation (Mean ± SD). A statistical comparison between all the treatments was performed. Data with asterisks (*) indicate a statistical difference (* *p* < 0.05) between the treatments compared to the untreated control (naive). The symbol above the bars (#) represents a statistically significant difference (# *p* < 0.05) between the marked treatments. (**B**) Cells treated with 10 µg/mL of different AgNPs sizes and the control. Images for the remaining AgNP concentrations and sizes can be found in the Supplementary Materials (Figure S4).
