# Supplementary material for: Mechanisms of Silver Nanoparticle Uptake by Embryonic Zebrafish Cells
Source: Nanomaterials (Basel). 2021 Oct 13;11(10):2699. doi: 10.3390/nano11102699 (PMC8541679; doi:10.3390/nano11102699)
Supplement: Supplementary file 1 [file nanomaterials-11-02699-s001.zip › nanomaterials-1307147-supplementary.pdf]

nanomaterials

Article

Mechanisms of Silver Nanoparticle Uptake by Embryonic Zebrafish Cells

Ana C. Quevedo, Iseult Lynch * and Eugenia Valsami-Jones

School of Geography, Earth and Environmental Sciences, University of Birmingham, Edgbaston, Birmingham B15 2TT, UK; AIC585@bham.ac.uk (A.C.Q.); E.ValsamiJones@bham.ac.uk (E.V.-J.)
* Correspondence: i.lynch@bham.ac.uk

Citation: Quevedo, A.C.; Lynch, I.; Valsami-Jones, E. Mechanisms of Silver Nanoparticle Uptake by Embryonic Zebrafish Cells. *Nanomaterials* **2021**, *11*, 2699. https://doi.org/10.3390/nano11102699

Academic Editors: Ernesto Alfaro and Ayse Basak Engin

Received: 4 July 2021
Accepted: 8 October 2021
Published: 13 October 2021

Publisher's Note: MDPI stays neutral with regard to jurisdictional claims in published maps and institutional affiliations.

Copyright: © 2021 by the authors. Licensee MDPI, Basel, Switzerland. This article is an open access article distributed under the terms and conditions of the Creative Commons Attribution (CC BY) license (https://creativecommons.org/licenses/by/4.0/).

1. Characterisation of the AgNPs

Characterisation of the AgNPs by Transmission electron microscopy (TEM). Results can be found as Figure S1 in the publication Quevedo et al., 2021.

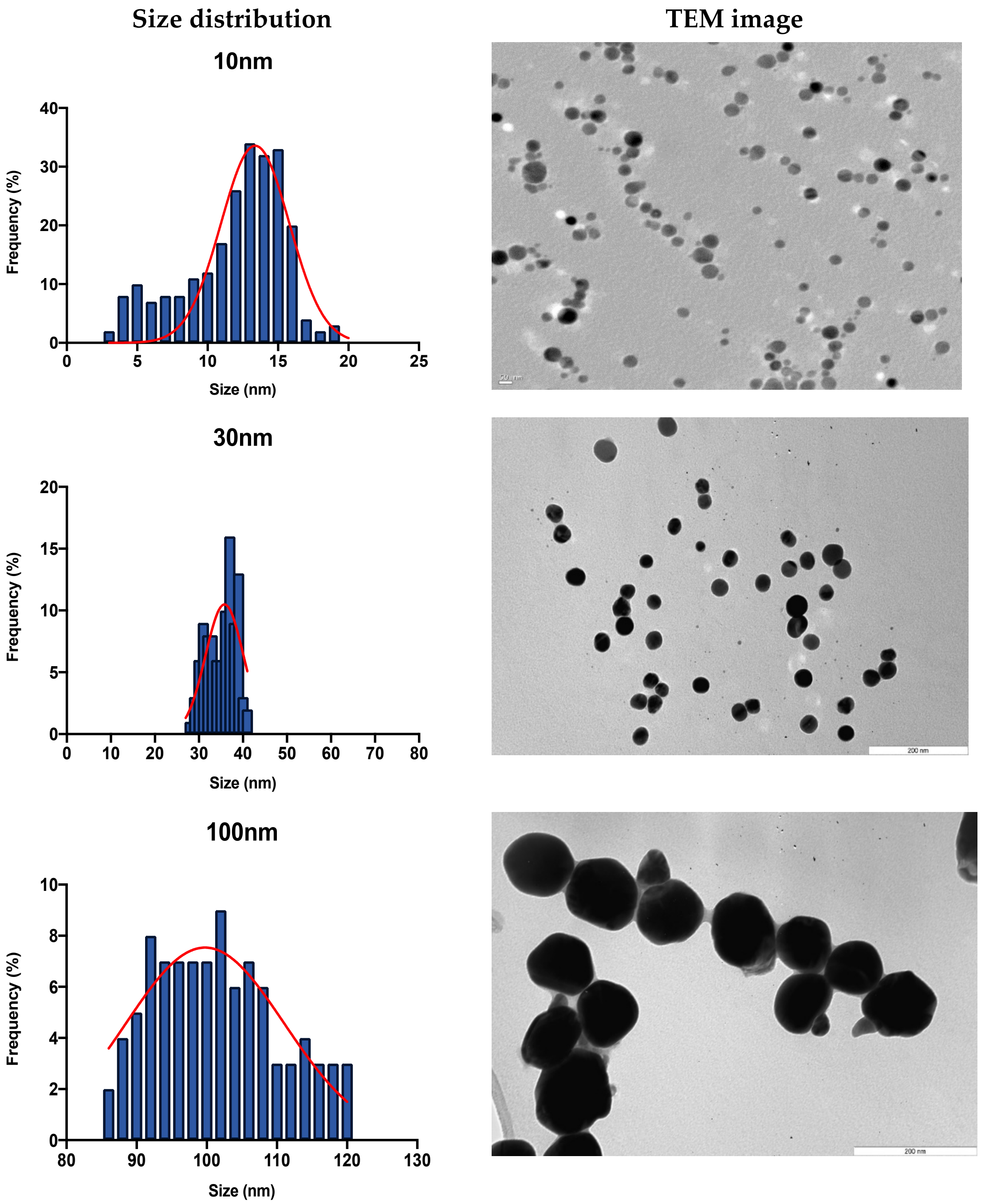

Figure S1. Characterisation of the AgNPs by TEM.

The characterisation of the AgNPs for this study was also performed by different methods including Dynamic light scattering (DLS), and Ultraviolet–visible spectroscopy (UV-vis). Polydispersity index and zeta potential were evaluated by DLS.

Table S1. Characterisation of the AgNPs. Characterisation in Ultrapure Water (UPW) and Complete Culture Media (CCM) (DMEM-F12 supplemented with 10% Foetal Bovine Serum, FBS) at 0 and 24 hours. Results represent the mean of three individual replicates and their standard deviation.

**10 nm**				
	**Water 0 hours**	**Water 24 hours**	**CCM 0 hours**	**CCM 24 hours**
**Hydrodynamic size (DLS)**	66.36 ± 0.7	68.05 ± 1.29	69.24 ± 4.96	128.54 ± 2.56
**PDI**	0.42 ± 0.007	0.42 ± 0.009	0.48 ± 0.004	0.505 ± 0.001
**Zeta potential (mV)**	-17.73 ± 0.61	-20.33 ± 2.02	-7.48 ± 0.53	-12.23 ± 0.85
**UV-Vis (abs)**	1.283 ± 0.000	0.745 ± 0.000	0.952 ± 0.000	0.552 ± 0.000
**30 nm**				
	**Water 0 hours**	**Water 24 hours**	**CCM 0 hours**	**CCM 24 hours**
**Hydrodynamic size (DLS)**	77.01 ± 1.33	81.48 ± 1.07	74.81 ± 1.61	160.70 ± 21.69
**PDI**	0.14 ± 0.002	0.14 ± 0.01	0.30 ± 0.003	0.42 ± 0.06
**Zeta potential (mV)**	-28.60 ± 0.65	-30.16 ± 2.46	-8.67 ± 0.09	-10.46 ± 0.88
**UV-Vis (abs)**	0.698 ± 0.010	0.403 ± 0.011	1.093 ± 0.003	1.072 ± 0.000
**100 nm**				
	**Water 0 hours**	**Water 24 hours**	**CCM 0 hours**	**CCM 24 hours**
**Hydrodynamic size (DLS)**	129.66 ± 1.62	127.1 ± 1.60	138.13 ± 1.06	182.06 ± 4.25
**PDI**	0.06 ± 0.01	0.03 ± 0.011	0.05 ± 0.01	0.14 ± 0.01
**Zeta potential (mV)**	-51.43 ± 1.32	-23.46 ± 0.56	-8.09 ± 0.28	-10.03 ± 1.41
**UV-Vis (abs)**	0.318 ± 0.037	0.293 ± 0.001	0.617 ± 0.002	1.195 ± 0.003

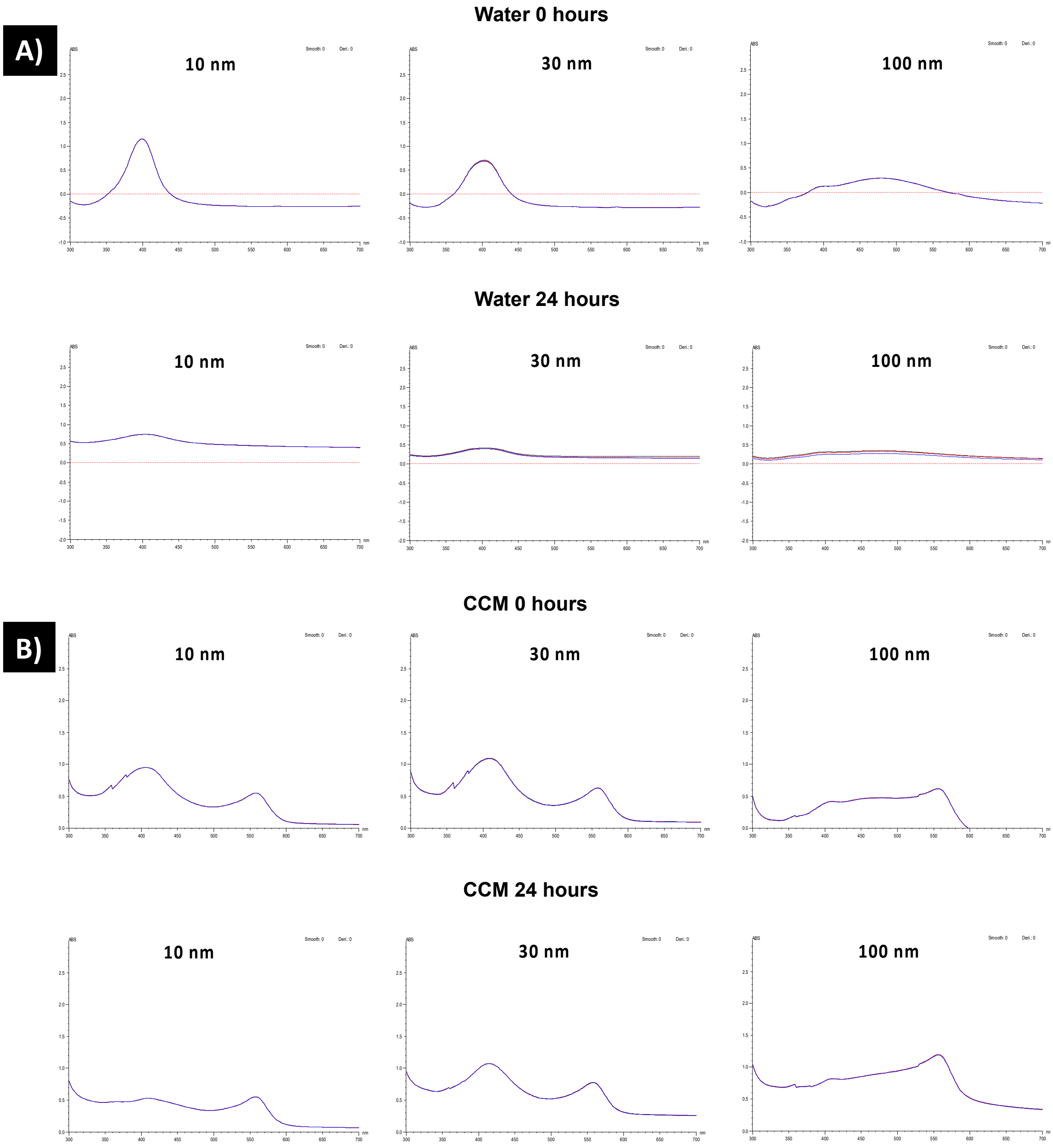

Figure S2. UV-vis images. UV-Vis recordings of the 10, 30 and 100 nm AgNPs at 0 and 24 hours in (A) UPW and (B) CCM.

2. Visualisation of the Intracellular AgNPs

Cells were treated with the highest concertation of 10, 30 and 100 nm AgNPs (10 µg/mL) for 24 hours, then cells were processed (see materials and methods) for transmission electron microscopy (TEM) analysis to assess the size of the internalised AgNPs. Images of the ultramicrotome sections were recorded using JEOL 1200EX 80kV and JEOL 1400EX 80kV microscopes. The intracellular size of the AgNPs in the vacuoles was calculated using Image J software. A total of 20 NPs were counted for each AgNP size to calculate their average size and standard deviation. Similarly, the diameter of 3 vacuoles (with AgNPs inside) was used to calculate their average size and standard deviation following internalisation of AgNPs of each size.

3. Intracellular Localisation of AgNPs

The intracellular NPs were analysed by manually selecting 15 NPs inside vesicles in the TEM images. Then, the diameter of the NPs was calculated using ImageJ V2.0.0 free software. First the bar scale was set according to the TEM image, then the diameter of 15 NPs was calculated. Measurements were processed in excel using to obtain the percentage (%) of reduction by comparing the intracellular diameter against the core size measurements by TEM (sample prepared in suspension and without cells). The same principle to obtain the diameter size was followed for three vesicles.

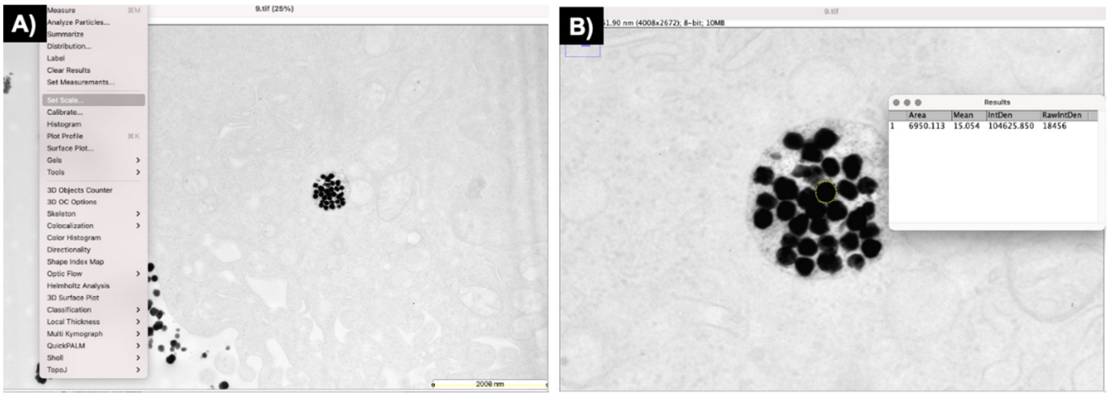

Figure S3. Analysis of intracellular AgNPs and vesicles.

Table S2. Intracellular AgNP size determined by TEM. Cells were treated with 10 μg/mL of different AgNPs sizes for 24 hours and processed for TEM analysis. The NPs inside the vesicles were counted to calculate their average size and standard deviation. The diameter of 3 vacuoles (with AgNPs inside) were used to calculate their average size and standard deviation following internalisation of each AgNP size.

AgNPs size	TEM size (nm)	Intracellular NPs		Vacuole diameter
		Size (nm)	Reduction (%)	nm
**10 nm**	13 ± 2.4	5.50 ± 3.56	57.62	717.46 ± 78.57
**30 nm**	34 ± 2.8	27.52 ± 4.20	19.04	678.87 ± 2.17
**100 nm**	101 ± 9.2	96.78 ± 8.36	4.12	697.86 ± 53.76

4. Cellular Uptake Determined by Inductively Coupled Plasma Mass Spectrometry (ICP-MS)

The total intracellular concentration of Ag^+ was measured by ICP-MS after different exposure times. The normalised mass concentration (μg/mL) results were recalculated to obtain the total Ag^+ per cell.

Table S3. Results for the total Ag^+ concentration per cell (pg/cell) determined by ICP-MS. Cells were treated with different concentrations and sizes of AgNPs for 2 and 24 hours. Then, the mass results (µg/mL) obtained by ICP-MS were re-calculated as Ag^+/cell based on the cell viability (no. of cells) recorded (LDH assay) at the AgNP concentration exposed (2.5, 5 and 10 µg/mL) from a total number of 5×10^5 cells seeded. Then Ag^+/cell results were normalized to an equal cell number of 100,000 cells and transformed to pg/cell. Results show the mean of three individual replicates and their standard deviation.

	AgNP initial concentration		
**AgNP size**	**2.5 µg/mL**	**5 µg/mL**	**10 µg/mL**
	**2 hours**		
**10 nm**	0.47 ± 0.09	0.48 ± 0.01	0.25 ± 0.09
**30 nm**	0.05 ± 0.06	0.01 ± 0.01	0.03 ± 0.01
**100 nm**	0.07 ± 0.02	0.03 ± 0.006	0.03 ± 0.01
	**24 hours**		
**10 nm**	0.54 ± 0.09	2.40 ± 0.08	8.97 ± 1.41
**30 nm**	0.27 ± 0.07	0.54 ± 0.02	3. 68 ± 0.29
**100 nm**	0.67 ± 0.05	1.71 ± 0.20	5. 92 ± 0.20

LDH Assay

The methodology used to select a representative set of exposure concentrations is described in Quevedo et al., 2020. Briefly, ZF4 cells were seeded in 96-well plates at a density of 8,000 cells/mL using DMEM/F12 supplemented with 10% FBS, and 1% penicillin and streptomycin at 28 °C, 5% CO_2. 24 hours post seeding, cells were treated with 5, 10, 20, 30, 40 and 60 µg/mL of AgNPs and incubated for 24 hours. Lactate dehydrogenase (LDH) levels (LDH kit Promega) were analysed after the incubation period using a modified protocol as described by (Ali-Boucetta, Al-Jamal et al. 2011). Briefly, the cell medium was aspirated and replaced with 110 µL of 0.9% lysis solution and incubated for 45 minutes at 28°C. Lysates were collected, transferred into 1.5 mL Eppendorf tubes and centrifuged at 13,500 rpm (Eppendorf, 5430R) for 5 minutes; 50 µL of the cell lysate was transferred into individual wells of 96 well plates, followed by addition of 50 µL of reconstituted substrate mix (LDH kit Promega, G1780), covered with foil and incubated at room temperature for 15 minutes. Finally, 50µL of Stop solution (LDH kit Promega) was added; the absorbance was immediately recorded using a plate reader at 492 nm (OMEGA). All experiments were performed in triplicate, for each AgNPs size (10, 30 and 100nm) and $AgNO_3$. Results were calculated based on the percentage of cell survival using the formula:

$$\text{Percentage of survival} = [\text{sample absorbance}/\text{mean control absorbance}] \times 100 \quad (1)$$

Finally, 2.5, 5 and 10 µg/mL were selected as low, medium and high (EC_{50}) AgNP concentrations for all subsequent exposures.

5. Inhibition of the Cellular Uptake Pathways

The inhibition of cellular uptake of the AgNPs was assessed by determining the total silver uptake by ICP-MS following inhibition of the selected pathway. First, the pharmaceutical inhibitor concentrations were confirmed by inhibition of the respective controls known to be internalised by that specific pathway using confocal microscopy. Then, the selected inhibitor concentrations were used to inhibit the desired pathway, and the total amount of Ag^+ was assessed by ICP-MS and compared to that in the untreated control. Any difference in internalised Ag content is due to a reduction of the specific internalisation pathway. Chlorpromazine is an inhibitor of the clathrin-mediated uptake pathway, while Genistein interferes with caveolae-mediated endocytosis and wortmannin inhibits macropinocytosis.

Table S4. Inhibition of uptake pathways and resulting changes in AgNP uptake and internalisation. After the inhibition of the desired pathway, cells were treated with the different concentrations and sizes of AgNP for 2 hours. Then, the total Ag^{+} was quantified by ICP-MS. The results are normalized to percentage against their control (no inhibitor). The results represent the mean of three individual replicates and their standard deviation.

AgNP concentration	Control (no inhibitor)	Chlorpromazine (clathrin-mediated)	Genistein (caveolae-mediated)	Wortmannin (macro pinocytosis)
		**10 nm**		
**2.5 µg/mL**	100 ± 2.76	98.70 ± 2.24	70.38 ± 3.67	54.48 ± 19.30
**5 µg/mL**	100 ± 6.38	93.10 ± 8.34	30.71 ± 5.13	27.61 ± 2.12
**10 µg/mL**	100 ± 5.53	39.54 ± 4.67	6.73 ± 1.12	7.27 ± 1.05
		**30 nm**		
**2.5 µg/mL**	100 ± 2.76	77.38 ± 17.57	17.90 ± 4.03	63.16 ± 16.47
**5 µg/mL**	100 ± 6.38	86.53 ± 8.32	30.16 ± 2.35	58.63 ± 30.44
**10 µg/mL**	100 ± 5.53	100.7 ± 0.75	31.68 ± 7.24	33.67 ± 3.60
		**100 nm**		
**2.5 µg/mL**	100 ± 2.76	35.44 ± 10.24	15.58 ± 5.91	27.70 ± 11.79
**5 µg/mL**	100 ± 6.38	35.09 ± 3.32	14.35 ± 6.5	42.99 ± 25.20
**10 µg/mL**	100 ± 5.53	39.04 ± 4.69	15.22 ± 2.71	66.06 ± 14.31

6. Early Endosome Induction (EEI)

Table S5. Early endosome induction. Cells were exposed to different concentrations and sizes of AgNPs for 2 and 24 hours, then the intensity of red fluorescent protein was recorded by florescence plate reader and results normalised to percentage (%) against naïve. Results show the average of three individual replicates and their standard deviation.

AgNPs size	Naive	2.5 µg/mL	5 µg/mL	10 µg/mL
		**2 hours**		
**10 nm**	0.00 ± 0.00	0.205 ± 0.111	0.178 ± 0.034	0.240 ± 0.079
**30 nm**	0.00 ± 0.00	0.225 ± 0.065	0.275 ± 0.060	0.277 ± 0.123
**100 nm**	0.00 ± 0.00	0.256 ± 0.105	0.253 ± 0.058	0.232 ± 0.054
		**24 hours**		
**10 nm**	0.00 ± 0.00	0.097 ± 0.078	0.058 ± 0.022	0.126 ± 0.060
**30 nm**	0.00 ± 0.00	0.123 ± 0.067	0.133 ± 0.048	0.171 ± 0.115
**100 nm**	0.00 ± 0.00	0.173 ± 0.055	0.183 ± 0.043	0.177 ± 0.031

7. Autophagy Response

The autophagy response was assessed by confocal microscopy. Images were taken and the intensity of fluorescein isothiocyanate (FITC), which has a fluorescence excitation/emission of 499/521 nm (autophagosome marker, Cell Meter™assay kit), in cells after the exposure to AgNPs was recorded.

Table S6. Autophagy results. Cells were treated with different concentrations and sizes of AgNPs for 24 hours. Then, the mean intensity of the autophagosome marker FITC was recorded by image J. The intensity results after 24 hours of to the AgNPs exposure were normalized to percentage (%) against naïve. The results represent the mean of three individual replicates and their standard deviation.

AgNPs size	Naive	2.5 µg/mL	5 µg/mL	10 µg/mL
**10 nm**	0.0 ± 0.0	2.47 ± 0.89	1.24 ± 1.4	2.17 ± 1.65
**30 nm**	0.0 ± 0.0	1.39 ± 0.37	0.19± 0.12	0.65 ± 0.53
**100 nm**	0.0 ± 0.0	0.32 ± 0.07	0.28 ± 0.37	0.11 ± 0.59

Confocal Microscopy Images of Autophagy Induction

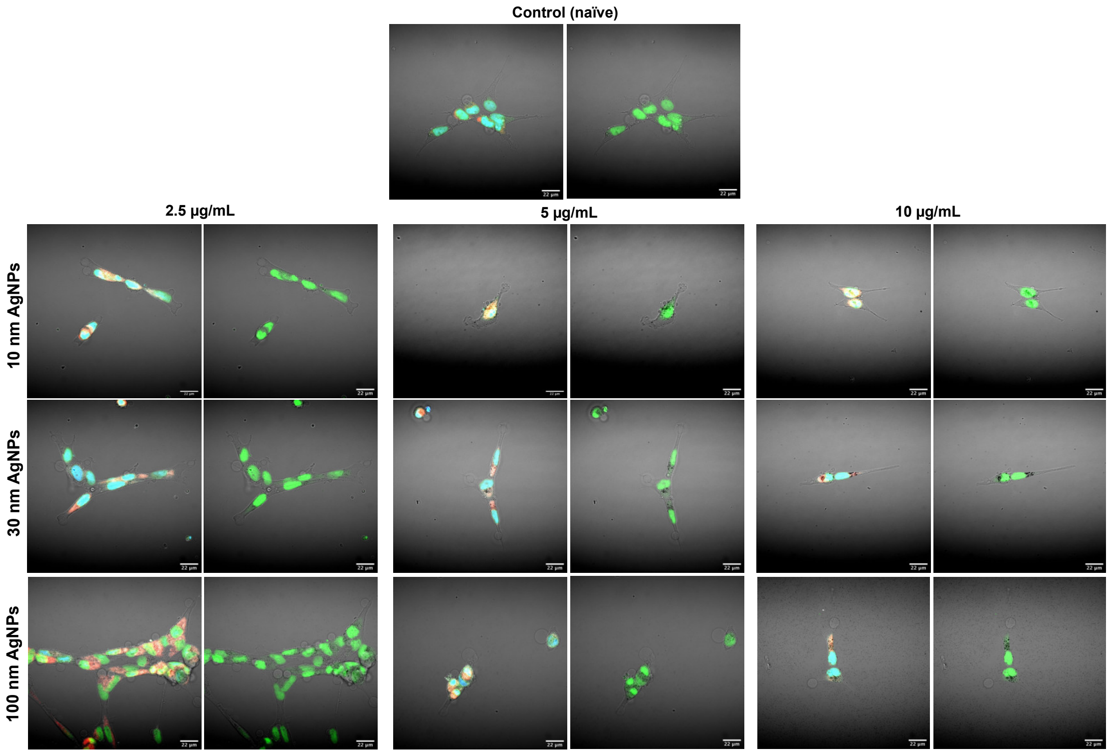

Figure S4. Images of autophagy induction. ZF4 cells were exposed for 24 hours to AgNPs at different concentrations and sizes. Then, images were taken with a 60X objective lens by NIKON A1R 808 microscope. The images (left) show the overlay of three different stains used for cell localisation: blue for nucleus, red for lysosomes and green is the autophagy dye. All the images (except control) also have an overlay of the 488-channel intensity for the AgNPs (white dots).

8. Protein Corona Identification

8.1. Protein Corona Isolation by SDS-PAGE Gel

The protein corona was isolated by centrifugation as follows: Cells were seeded in six-well plates at a density of 5 × 10^5 cells per well in 2 mL of DMEM/F12 supplemented with 10% FBS (known as Complete Culture Media (CCM)) and 1% penicillin and streptomycin at 28 °C and 5% CO_2. After 24 hrs, cells were treated with 2.5, 5 and 10 µg/mL of AgNPs of sizes 10, 30 and 100 nm for 2 or 24 hours, or only with CCM for 24 hours. After the exposure time, the cell medium containing the AgNPs that had not been taken up by the cells was removed, placed in 2mL Eppendorf tubes and centrifuged at 14,000 rpm for 20 minutes at 4°C. Then, the supernatant was discarded, and the AgNP pellet was washed with 1 mL of PBS. The washing step was repeated three times. The pellet was eluted with 100 µL of SDS 4x Laemmli sample buffer (Sigma, S3401) and incubated at 95°C for 5 minutes. The eluted corona was gently transferred into a fresh tube and stored at −20°C for future analysis. The isolated proteins were run on a 12.5% polyacrylamide gel electrophoresis (PAGE) at 170 V for 1.5 hours, then the gel was carefully washed with 10 mL of water and stained with 25% Coomassie blue staining at 37°C overnight. Afterwards, the gel was de-stained for 24 hours. Images were recorded by Gel Doc™ EZ Imager - Bio-Rad, using the white tray and selecting the protein analysis by Coomassie blue option in the software.

Bands confirming the presence of proteins for the highest AgNP concentration (10 mg/mL) and sizes (10, 30 and 100 nm) were cut and sent for protein/peptide identification at the Advanced Mass Spectrometry Facility in the School of Biosciences at the University of Birmingham. The results were compared against the Zebrafish database.

Table S7. Analysis of the AgNP corona compositions. Proteins identified by mass spectrometry in the 10, 30 and 100 nm AgNP sizes following exposure to ZF4 cells for 24 hours. Results represent the summary of the proteins found in the isolated protein corona for the three AgNPs sizes following a search in the zebrafish proteome database (Uniprot).

ACCESSION	DESCRIPTION	MW [KDA]
**F1QGZ6**	Maternal embryonic leucine zipper kinase OS=Danio rerio OX = 7955 GN = melk PE = 2 SV = 1	76.8
**Q90416**	Retinoic acid receptor RXR-gamma-A OS = Danio rerio OX = 7955 GN = rxrga PE = 2 SV = 2	48.7
**P79725**	Swelling dependent chloride channel, ICln OS = Danio rerio OX = 7955 GN = clns1a PE = 4 SV = 1	27.4
**Q6K197**	p65 transcription factor OS = Danio rerio OX = 7955 GN = rela PE = 2 SV = 1	51.1
**Q1XA84**	Ataxin-3 (Fragment) OS = Danio rerio OX = 7955 GN = atxn3 PE = 2 SV = 1	34.5
**P50541**	Max-interacting protein 1 OS = Danio rerio OX = 7955 GN = mxi1 PE = 2 SV = 1	27.6

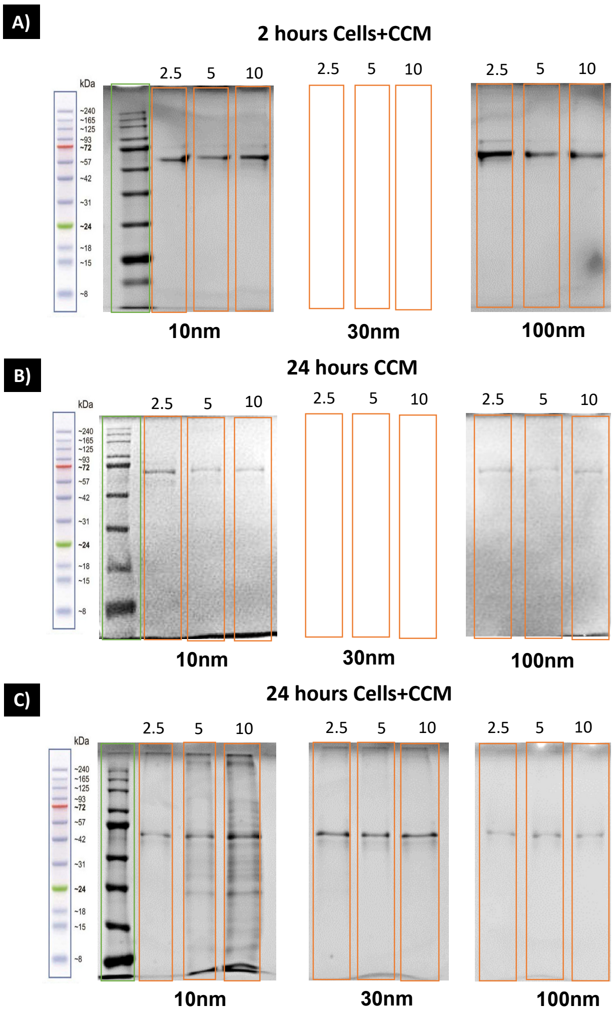

Figure S5. Coomassie blue staining of PAGE gels for isolated protein corona. ZF4 cells were treated with different concentrations of AgNPs (2.5, 5 and 10 µg/mL) and sizes (10, 30 and 100 nm) for 2 or 24 hours. The lanes outlined in green represent the protein ladder and the orange lanes correspond to the samples. The numbers above the bands show the AgNPs concentrations used in µg/mL. A) AgNPs treatments prepared in CCM (DMEM-F12 supplemented with 10% FBS) and incubated with ZF4 cells for 2 hours. B) AgNPs treatments in CCM only (no cells) incubated for 24 hours so indicating the FBS corona. C) AgNPs treatments prepared in CCM and incubated with ZF4 cells for 24 hours, indicating the evolution of the corona as the ZF4 cells condition the medium.

8.2. Secreted Proteins

To further understand the role of the ZF4 cell secreted proteins in the formation of the protein corona, the total protein content of AgNPs treatments in CCM and serum-free medium (SFM) was quantified. The experiment was designed to mimic the real conditions in which most of the experiments in this study were performed (NPs and proteins free in solution). Here, cells were seeded in 6 well plates at a density of 5 × 10^5 cells/mL using DMEM/F12 supplemented with 10% FBS and 1% penicillin and streptomycin at 28 °C and 5% CO_2. After 24hrs, cells were treated with 2.5, 5 and 10 µg/mL of 10, 30 and 100 nm AgNPs in CCM. After 3, 6, 12 and 24 hours the volume in the wells (1mL) with the NP treatments was gently resuspended, then a sample of 20 µL (under sterile conditions) was taken and placed in 96 well plates. Protein quantification was immediately performed using a Pierce™ BCA Protein Assay Kit following the supplier's protocol. A control with naïve cells in CCM was also included to demonstrate changes in the protein concentrations across the time points.

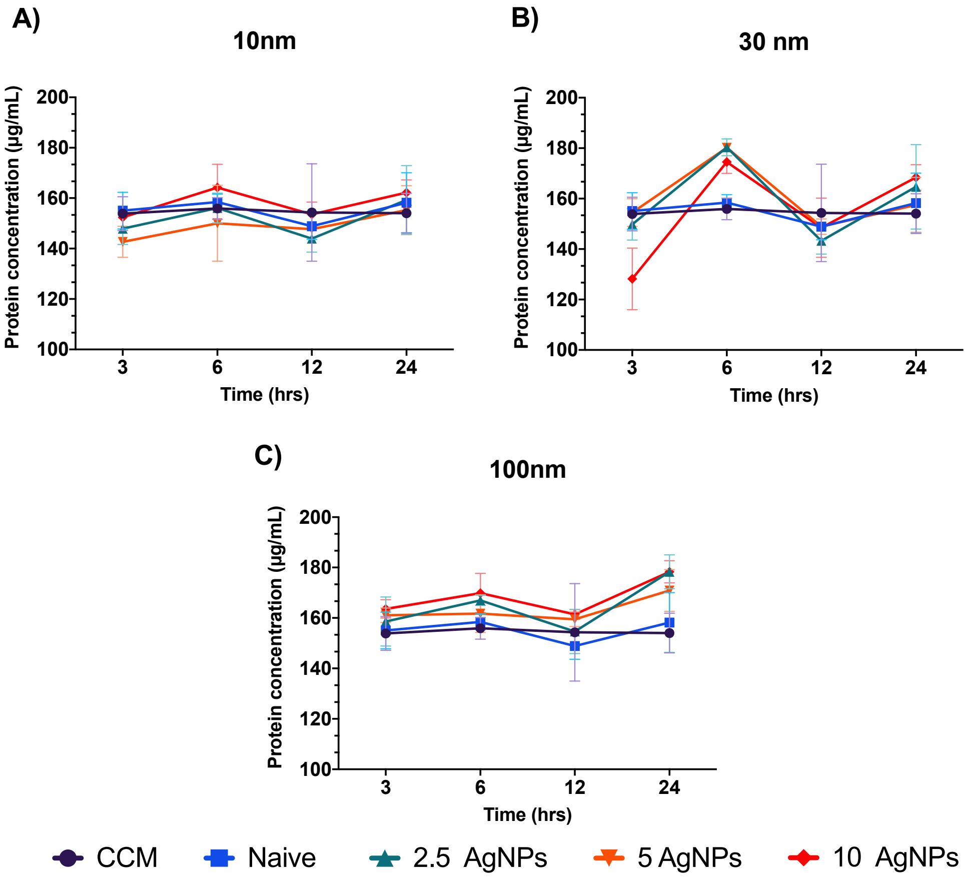

Figure S6. Proteins secreted by ZF4 cells in response to AgNPs exposure in medium containing 10% FBS. ZF4 cells were exposed to 2.5, 5, and 10 µg/mL of AgNPs prepared in CCM (10% FBS), and the protein content in the medium was measured by BCA assay at 3, 6, 12, and 24 hours, as a means to assess the cellular response to NP exposure and to quantify the amount of protein secreted by the ZF4 cells in response to increasing exposure time. A control of cells (without treatment) in CCM only was included to compare with the NPs' treatments.

References

1. Ali-Boucetta, H.K. T.; Al-Jamal, K. H.; Muller, S.; Li, A.E.; Porter, A.; Eddaoudi, M.; Bianco, P.A.; Kostarelos, K. Cellular uptake and cytotoxic impact of chemically functionalized and polymer-coated carbon nanotubes. *Small* **2011**, *7*, 3230–3238.
2. Quevedo, A.C.; Lynch, I.; Valsami-Jones, E. Silver nanoparticle induced toxicity and cell death mechanisms in embryonic zebrafish cells. *Nanomaterials* **2021**, manuscript submitted for publication.
